# Mental health penalties of having a child: findings from the China family panel studies

**DOI:** 10.1038/s44184-023-00026-x

**Published:** 2023-05-15

**Authors:** Xinjie Shi, Yu Shen

**Affiliations:** 1 China Academy for Rural Development, School of Public Affairs, Zhejiang University, Hangzhou, China; 2 Research Center for Common Prosperity, Future Regional Development Laboratory, Innovation Center of Yangtze River Delta, Zhejiang University, Jiaxing, China; 3 Center for Common Prosperity of Zhejiang University & Huzhou City, Hangzhou, China; 4 Institute for Common Prosperity and Development, Zhejiang University, Hangzhou, China; 5 School of Economics, Nanjing University of Finance and Economics, Nanjing, China

**Keywords:** Health care, Economics, Sociology

## Abstract

In recent years, the birth rate in China has rapidly declined. While much research has been done on the penalties in earnings that women incur when they fall behind men in the labor market due to childbirth, there has been little to no research on the mental health effects. This study addresses the gap in current literature by examining the mental health penalties that women experience after having a child in comparison to men. We applied econometric modeling to data collected from China Family Panel Studies (CFPS) and found that women experienced a significant, immediate, and long-run decline (4.3%) in life satisfaction after their first child, while men were unaffected. We also found that women experienced a significant increase in depression after their first child. This suggests mental health penalties since the mental health risk proxied by these two measurements is only significant for women. This is likely related to child penalties in labor market performance and childbirth-related physical health issues. When countries adopt multiple tools to stimulate the birth rate for economic growth, they must consider the implicit burden on women—especially the long-term negative effects on mental health.

## Introduction

Achieving gender equality is one of the United Nation’s Sustainable Development Goals (SDG). It is an increasingly important avenue of research and area in need of renewed policy implementation as we work towards a shared prosperity globally. An important dimension of gender disparity stems from a women’s ability to conceive and carry a child. Each year, around 210 million women become pregnant^1,2^. Despite the global effort to provide adequate maternity care, maternal mortality remains high, with approximately 303,000 dying in 2015^3^. This figure was likely even higher after the coronavirus disease (COVID-19) in 2020. The challenges are much more severe in low-and middle-income countries (LMIC) than in high-income countries (HIC)^4^. There is a rapidly growing body of literature on the drivers of maternity care^5^, the dynamic burden of poor maternal health^2^, and further calls to action^6–8^. However, these studies have not adequately examined the long-term impacts of childbirth on women.

There is compelling evidence for child penalties, which is widely defined as the extent to which women fall behind men due to having a child. Extant studies have addressed labor-related child penalties, highlighting the negative impact of maternity on women’s labor market opportunities^9^, income^10^, and intra-household bargaining power^11–13^. However, child penalties are far more than merely labor-related as they are also related to ill-health, of which there is very limited research. Mental illness in women is a popular public health problem. It is well known that women are more likely to have severe mental health issues—including depression and anxiety—than men^14,15^. This is highly associated with biological differences in hormone profiles, which suggests that women have a higher probability of depression during times of reproductive endocrine changes such as postpartum^15^. While research has shown that childbirth may have short-term negative consequences for a woman’s physical health^16,17^, there have been far fewer studies focusing on the long-term effects on mental health. Physical health issues and poor labor market performance may compound their mental health problems and lead to even more personal and familial repercussions^18,19^. Studies have shown that mental disorders (e.g., depression) are a major risk factor contributing to suicidal behavior—one of the three leading causes of death in the productive age group^20^. In light of this, we aim to examine whether childbirth contributes to a higher occurrence of mental illness in women who experience childbirth. For comparative purposes, we show whether this is the case for men as well.

For decades, the Chinese population count has been globally one of the largest. However, in contrast, the birth rate has begun to decline: from approximately 12.02 million in 2020 to 10.62 million in 2021. We propose that child penalties might possibly explain the lack of desire in Chinese women to reproduce. Studies find that mental health issues are quite prevalent in China, greater than 17% and that mood and anxiety disorders are more prevalent in women^21^. These facts make China an appropriate setting for studying child penalties in mental health. In this study, we examined child penalties in relation to women’s (compared to men’s) mental health in China using a sample of individuals aged 16–50 years from the China Family Panel Studies (CFPS), including those in 2010, 2012, 2014, 2016, and 2018. We investigated whether—and if so, to what extent—childbirth affected individual’s mental health and examined how this effect differed between men and women. Furthermore, we analyzed whether the case was identical for women with different socio-demographic characteristics and how it was associated with child penalties in labor market performance and physical health status.

## Methods

### Sample description

The CFPS is a nationally representative, annual longitudinal survey conducted by the Institute of Social Science Survey (ISSS) of Peking University, China. The CFPS sample covers 25 provinces and autonomous regions, and the target sample size is 16,000 households. CFPS carried out preliminary and follow-up test surveys in Beijing, Shanghai, and Guangdong in 2008 and 2009, and officially conducted visits in 2010. The respondents include all family members in the sample households. All baseline family members and their children defined by the 2010 baseline survey will be the genetic members of CFPS and become permanent tracking objects. Officially launched in 2010, this survey collects individual-, family-, and community-level information with a specific focus on economic activities, family background, migration, health, and education. With a wide range of domains for families and individuals from 25 provinces of China, we primarily used the survey data collected in 2010, 2012, 2014, 2016, and 2018, which provided consistent mental health variables. Our analyses included 11,520 women (52%) and 10,495 men (48%) interviewed in the five waves.

All the participants (or their legal representatives) gave their informed consent to participate in the baseline and follow-up surveys. This project was approved by the Biomedical Ethics Committee of Peking University, Beijing, China (IRB00001052–14010).

### Measures of health

We used life satisfaction as an indicator of main mental health. Each wave of CFPS asked the respondent: “How satisfied are you with your life?” The response options ranged from 1 (*not satisfied at all*) to 5 (*very satisfied*). Life satisfaction can be defined as the subjective measurement of personal well-being, and reflects the degree to which an individual’s experience matches their long-term expectations for life^22,23^. The related question in CFPS used to measure the degree of short-term depression is as follows: during the past month, how often did you feel so frustrated/depressed that nothing could cheer you up. The answer ranges from 1 to 4 which represents the frequency of this type of feeling: 1 (almost never), 2 (sometimes), 3 (often), 4 (most of the time).

Although this study focused on mental health, it also examined the differences in physical impact of childbirth on men and women. We measured physical health via three indicators: (1) presence of any chronic diseases, (2) improvement in physical health, and (3) hospitalizations in the previous year. As for the second indicator, the questionnaire asked participants: “How does your current health compare to one year ago?” Based on their self-reported answers, we constructed the dummy variables as 1 for “better” and 0 for “no change,” and “worse.”

### Measures of labor market performance and socio-demographics

We adopted four measures of labor market performance—an indicator for employment, hours worked per week, annual income and labor productivity (hourly wage)—to examine whether child penalties on mental health were associated with fewer labor market opportunities for women. As for the main socio-demographic variables for baseline regressions, we first control for years of schooling which are widely adopted as a human capital proxy associated with mental health^24^. We also control for present age and age at the time of having the first child. Living in an urban area or being a primary caregiver were used as indicators. The responsibility of taking care of children is more likely to be shared by extended family in urban China^25^.

### Statistical analysis

The descriptive statistics of the main variables for the full sample and for women and men respectively are presented in Table 1. For each respondent, we obtained information before and after the birth of the first child; we retained only those samples that had at least one child. On average, women (3.618) had a higher score for life satisfaction than men (3.493). However, the results using event studies below, imply that women’s mental health worsened after childbirth, while that of men did not. Moreover, women had a higher probability of hospitalization (9.9%) than men (4.3%). Additionally, women showed worse labor market performance than men, measured by the probability of being employed (0.670 versus 0.943), hours worked per week (48 versus 54), annual income (11,930 versus 24,050 yuan) and hourly wage (11.65 versus 17.68 yuan). Socio-demographics reveal that women, compared to men, are younger (aged 29 versus 31) and less educated (9.63 versus 10.00 years of schooling).

**Table 1:** Summary statistics.

Var Name	All		Female		Men		Difference between gender (Mean of Women-Mean of Men)
	Obs	Mean	Obs	Mean	Obs	Mean	
Mental health							
Life satisfaction	22,015	3.558	11,520	3.618	10,495	3.493	0.125***
Depression	21,981	1.637	11,500	1.684	10,481	1.585	0.098***
Physical health							
Chronic (Yes = 1)	22,002	0.060	11,513	0.060	10,489	0.061	−0.001
Improved Physical health status (Yes = 1)	22,006	0.107	11,514	0.107	10,492	0.108	−0.001
Hospitalized last year (Yes = 1)	22,005	0.072	11,514	0.099	10,491	0.043	0.056***
Labor market performance							
Employment (Yes = 1)	17,104	0.801	8,931	0.670	8,173	0.943	−0.273***
Hours worked per week	12,366	51.440	5,414	48.252	6,952	53.923	−5.671***
Hourly wage (Yuan)	11,604	15.076	5,015	11.654	6,589	17.681	−6.027***
Income (10 Thousand Yuan)	18,409	1.785	9,425	1.193	8,984	2.405	−1.212***
Demographics							
Age	22,015	30.224	11,520	29.227	10,495	31.319	−2.091***
Schooling year	22,015	9.807	11,520	9.634	10,495	9.997	−0.363***
Living in Urban areas (Yes = 1)	21,781	0.501	11,396	0.501	10,385	0.501	−0.001
Children mainly taken care by mom (Yes = 1)	19,630	0.768	10,394	0.787	9,236	0.747	0.040***
Age when having the first child	22,015	25.441	11,520	24.449	10,495	26.531	−2.082***
Number of sons	21,865	0.853	11,449	0.855	10,416	0.851	0.005
Number of children	22,015	3.057	11,520	3.078	10,495	3.033	0.045**
Other Potential Mechanisms							
Self-reported social status	21,976	2.716	11,490	2.712	10,486	2.721	−0.008
Co-residence with parents (Yes = 1)	17,639	0.435	7,981	0.153	9,658	0.668	−0.515***
Financial support (Yes = 1)	9,910	0.259	4,887	0.241	5,023	0.277	−0.036***

The scores of life satisfaction range from 1 to 5, with higher scores indicating more satisfaction with current life. The value of depression ranges from 1 to 4, with the higher score, representing the more serious depressive symptoms in the past month. The value of self-reported social status ranges from 1 to 5, with the higher score indicating the higher status. The co-residence with parents is equal to 1 if women live with parents, 0, otherwise. The financial support is equal to 1 if receiving financial support from parents, 0, otherwise. A *t* test was conducted to compare the difference of average value for each variable between women and men: *p < 0.1, **p < 0.05, and ***p < 0.01.

### Event study: modeling the effect of childbirth on mental health

We estimated the impact of childbirth on the mental health outcomes of men and women using the event study approach^9^. An event study is a statistical approach to examine the effect of an event (e.g., childbirth) on a specific outcome (e.g., mental health) by combining regression discontinuity techniques around the time of the event. The treatment (event) in this study is the arrival of the first child. We observed the parents’ mental health each year from 5 years before the arrival of the first child until 10 years later. We then constructed the event study design by adding a series of events dummies as the independent variables. With a reference event time (say the event -1 that has been widely adopted), we are able to identify the impact of treatment (the arrival of the first child) in a specific event compared to that in the reference event time. The detailed specifications are as follows:

1 $${Y}_{{it}}={\alpha }^{{\prime} }{{\boldsymbol{Dt}}}_{{it}}+{{\beta }^{{\prime} }{\boldsymbol{Dm}}}_{{it}}+{\lambda }^{{\prime} }{{\boldsymbol{Dn}}}_{{it}}+{v}_{{it}},$$

where ${Y}_{{it}}$ is the life satisfaction of individual *i* at event time *t*. ${{\boldsymbol{Dt}}}_{{it}}$ is the vector term, including a series of event time dummies, and t = 0 denotes the year of the first child’s arrival; t = −5 signifies 5 years before the year of arrival of first child, and t = 5 signifies 5 years after the year of arrival of the first child, respectively. Following the literature^26^, we omitted the dummy for t = −1 as reference, so that $\alpha$ measured the impact of children on mental health in a given year relative to the year before the first child (t = −1). ${{\boldsymbol{Dm}}}_{{it}}$ and ${{\boldsymbol{Dn}}}_{{it}}$ include age and year dummies to control for life cycle and time trends^9^. Additionally, we ran this specification separately for men and women who had at least one child.

### Mediation model

We adopted a mediation model^27^ to further explore the mechanisms through which having a child may affect mental health. The model comprises the following three equations:

2 $${Y}_{{it}}={\alpha }_{0}+{\alpha }_{1}D+{\alpha }_{2}{X}_{{it}}+{\varepsilon }_{{it}1},$$

3 $${Y}_{{it}}={\beta }_{0}+{\beta }_{1}D+{\beta }_{2}{M}_{{it}}+{\beta }_{3}{X}_{{it}}+{\varepsilon }_{{it}2},$$

4 $${M}_{{it}}={\gamma }_{0}+{\gamma }_{1}D+{\gamma }_{2}{X}_{{it}}+{\varepsilon }_{{it}3},$$

where ${Y}_{{it}}$ is the same as above; D is a dummy indicating the status of childbirth, which is equal to 1 for the year of and after the first child and 0 otherwise. ${M}_{{it}}$ denotes mediation variables. ${X}_{{it}}$ denotes a set of control variables, and ${\varepsilon }_{{it}}$ denotes the error term. Our analysis includes four steps: (1) we estimate Eq. 2 to test whether ${\alpha }_{1}$ is significant, and if it is, we move to the second step; (2) we estimate Eq. 4, expecting ${\gamma }_{1}$ to be significant; (3) we estimate Eq. 3 to test whether ${\beta }_{2}$ is significant. If so, we can conclude that ${M}_{{it}}$ plays a role of mediation through which D affects ${Y}_{{it}}$; (4) we further test the significance of ${\beta }_{1}$. If it is insignificant, ${M}_{{it}}$ plays a role of full mediation; if it is significant, ${M}_{{it}}$ plays a role of partial mediation.

### Reporting summary

Further information on research design is available in the Nature Research Reporting Summary linked to this article.

## Results

### Mental health penalties of having a child

**Fig. 1:**
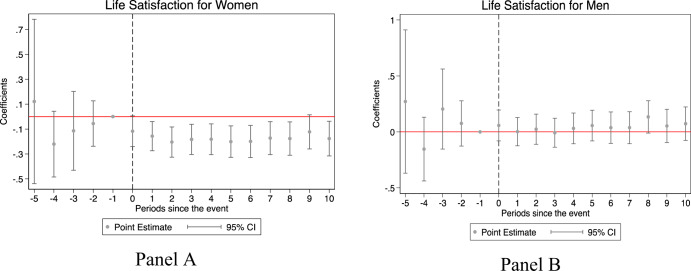
Changes in life satisfaction for women and men. **A** Life satisfaction for women. **B** Life satisfaction for men. Each dot represents a regression coefficient, and the corresponding vertical line (error bar) represents the 95% confidence interval. Schooling years, age-fixed effects, city-fixed effects, year-fixed effects, province year-fixed effects, the year of survey fixed-effects, and the month of survey fixed-effects are included. When the 0 from y axis is not covered by the vertical line, it signifies that the coefficient is significant and different from zero. The dashed gray line in event 0 signifies the year of arrival of the first child. The event-time dummy for t = −1 was omitted as the reference.

Figure 1 shows the effects of parenthood on the life satisfaction of women and men during different periods, respectively. Each dot indicates the impact at event time t (relative to event time -1) based on the above specification. The life satisfaction of men and women was not statistically different to that in event time −1; however, women experienced a significant, immediate, and long-term drop in life satisfaction after having their first child and had not recovered in 10 years. Men, on the contrary, were essentially unaffected.

To further examine the magnitude of these changes, we present the regression results corresponding to Fig. 1 in Table 2. Women’s life satisfaction score dropped by −0.157 (95% CI: −0.274 to −0.039) immediately after childbirth and persisted to −0.176 (95% CI: −0.316 to −0.037) for over 10 years. Considering the average score of life satisfaction, the percentage changes are −4.3% (−0.157/3.618) and −4.9% (−0.176/3.618), respectively. We repeated the regression for men before and after having their first child. The results showed that the impact of childbirth on men’ life satisfaction was insignificant for almost all years. To check whether these changes between men and women were statistically significant, we performed a statistic test for all the differences of coefficients between men and women in each of the years using seemingly unrelated regressions, finding that they were insignificant (*p* > 0.1) before childbirth and significant from the year of childbirth (*p* = 0.058) to 8 years after childbirth (*p* = 0.001). These results suggest the implied child penalty between men and women in the long-term are as follows: women face mental health issues immediately after the arrival of the new baby. This effect was significant for women compared to men even after 8 years. Men on the contrary, did not seem to face any mental health issues.

**Table 2:** Impact of childbirth on life satisfaction for men and women.

	(1)	(2)	(3)	(4)
	Women	Men	Significance of difference	
u_b5	0.121	0.271	u_b5_chi2	0.105
	[−0.538,0.781]	[−0.369,0.911]	u_b5_p	0.746
u_b4	−0.221	−0.154	u_b4_chi2	0.118
	[−0.485,0.043]	[−0.439,0.131]	u_b4_p	0.731
u_b3	−0.114	0.204	u_b3_chi2	1.785
	[−0.431,0.202]	[−0.153,0.562]	u_b3_p	0.182
u_b2	−0.056	0.076	u_b2_chi2	0.951
	[−0.238,0.126]	[−0.126,0.278]	u_b2_p	0.330
u_a0	−0.117^*^	0.058	u_a0_chi2	3.594
	[−0.240,0.005]	[−0.080,0.195]	u_a0_p	0.058
u_a1	−0.157^***^	0.002	u_a1_chi2	3.043
	[−0.274,−0.039]	[−0.125,0.129]	u_a1_p	0.081
u_a2	−0.204^***^	0.024	u_a2_chi2	6.413
	[−0.325,−0.083]	[−0.111,0.159]	u_a2_p	0.011
u_a3	−0.183^***^	−0.008	u_a3_chi2	3.742
	[−0.304,−0.062]	[−0.138,0.122]	u_a3_p	0.053
u_a4	−0.182^***^	0.031	u_a4_chi2	5.448
	[−0.305,−0.059]	[−0.106,0.169]	u_a4_p	0.020
u_a5	−0.201^***^	0.057	u_a5_chi2	7.883
	[−0.328,−0.075]	[−0.081,0.194]	u_a5_p	0.005
u_a6	−0.199^***^	0.038	u_a6_chi2	6.378
	[−0.328,−0.070]	[−0.103,0.178]	u_a6_p	0.012
u_a7	−0.173^**^	0.038	u_a7_chi2	4.880
	[−0.305,−0.040]	[−0.105,0.181]	u_a7_p	0.027
u_a8	−0.176^***^	0.134^*^	u_a8_chi2	10.406
	[−0.310,−0.042]	[−0.011,0.279]	u_a8_p	0.001
u_a9	−0.123^*^	0.053	u_a9_chi2	3.219
	[−0.260,0.015]	[−0.096,0.202]	u_a9_p	0.073
u_a10	−0.176^**^	0.073	u_a10_chi2	6.373
	[−0.316,−0.037]	[−0.077,0.223]	u_a10_p	0.012
Control	Y	Y		
Mean of Dep Var	3.618	3.493		
N	11,520	10,495		
R-sq	0.096	0.116		

Columns (1) and (2) show the results of event study for men and women, respectively. The independent variables are event time dummies, indexed such that u_bt means t years before the year of arrival of first child, and u_at means t years after the year of arrival of the first child, respectively. The event-time dummy at t = −1 was omitted, implying that the event-time coefficients measure the impact of children relative to the year just before the first childbirth. Individual controls included years of schooling and age fixed effects to control for life cycle trends. City-fixed effects, year-fixed effects, province-year-fixed effects, the year-of-survey-fixed effects, and the month-of-survey-fixed effects were included. To test whether the coefficients by subgroups are significantly different, we use the test drawing on seemingly unrelated regressions with the command *suest* in Stata. *p < 0.1, **p < 0.05, and ***p < 0.01, 95% confidence intervals are in brackets.

**Fig. 2:**
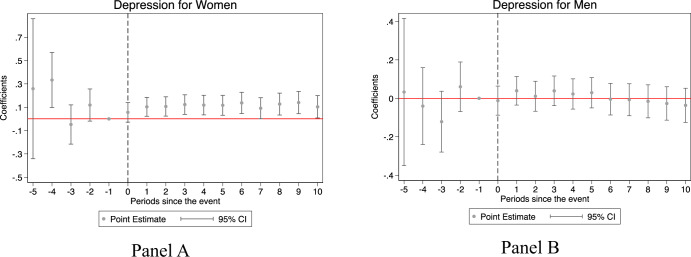
Changes in depression for women and men. **A** Depression for women, **B** Depression for men. Each dot represents a regression coefficient, and the corresponding vertical line (error bar) represents the 95% confidence interval. Schooling years, age-fixed effects, city-fixed effects, year-fixed effects, province year-fixed effects, the year of survey-fixed effects, and the month of survey-fixed effects are included. When the 0 from y axis is not covered by the vertical line, it signifies that the coefficient is significant and different from zero. The dashed gray line in event 0 signifies the year of arrival of the first child.

Some concerns remain. First, we were concerned that some key confounders influencing the mental health status were missing in the specifications above. For instance, the effect of preference for a son, and the age of women when the second child is born maybe pivotal. We conducted an additional robustness check on our main results when we took these factors into account (See Supplementary Tables 1 and 2). In additional to the controls in Table 2, column 1 further controls for the number of children and the number of sons, column 2 further controls for whether they have at least two children and their age when the last child was born. In column 3, we restrict the sample to those who have at least two children and control for their age when the second child was born. The main results are consistent with those in Table 2. Second, we were concerned that the unbalanced panel would lead to biased estimations. We further conducted two additional robustness checks, with the results shown in Supplementary Table 3. In columns (1) and (2), we excluded those that were surveyed once in 5 waves. In columns (3) and (4), we only included those who had the first child after 2010. The central results remain robust. Third, we added depression as the dependent variable. As defined by WHO, “mental health is a state of mental well-being that enables people to cope with the stresses of life, realize their abilities, learn well and work well, and contribute to their commurity” (https://www.who.int/news-room/fact-sheets/detail/mental-health-strengthening-our-response). Mental health conditions include mental disorders and psychosocial disabilities as well as other mental states associated with significant distress, impairment in functioning, or risk of self-harm.” In addition to life satisfaction, a positive aspect of mental health, depression is a major concern among Chinese people. We added this as a negative proxy of mental health to see whether childbearing penalty exists in depression by gender. This suggests mental health penalties since the mental health risk proxied by both life satisfaction and depression is only significant for women. Figure 2 and the corresponding Supplementary Table 4 show that women experienced a significant increase in depression after their first child, suggesting a worsened mental health status. Fourth, we also conducted robustness checks to examine whether the results are identical if sampling weights are adjusted. The results shown in Supplementary Table 5 identify child penalties for women rather than for men.

### Connection between child penalties, physical health status, and work performance

In this section, we show evidence with regard to child penalties in physical health and work performance, as these are likely to be associated with mental health penalties of having a child. Supplementary Fig. 1 and Supplementary Tables 6–8 show the physical health status before and after the arrival of the first child. For mothers and fathers, the probability of developing a chronic disease did not increase sharply after a child was born; however, the other two measures of physical health, including hospitalization experience and improved physical health, were affected by child penalties. A sharp increase was observed in the probability of reporting hospitalization in the first 2 years after childbirth for women, although it became insignificant after that; however, this was not the case for men throughout the 10-year postpartum. The gender disparities in the indicator for improved physical health were more profound, with women being negatively affected for 10 years after childbirth, while men were not.

Furthermore, we ran regressions to examine the impact of parenthood on work performance for women and men. The results in Supplementary Fig. 2 and Supplementary Tables 9–12 show that female labor supply and income (10,000 Yuan) dropped immediately after childbirth with the coefficients being −0.124 (95% CI: −0.195 to −0.053) and −0.565 (95% CI: −0.764 to −0.367), while male correspondence was essentially unaffected. The coefficient of event time 1 regarding hours worked is −2.959 (95% CI: −6.252 to 0.335) for women, suggesting that women’s hours worked per week decreased by 2.959 the first year after childbirth; this, however, is only significant at the 10% level. Child penalties in hourly wage as a measure of productivity are also identified for women.

### Heterogeneity in child penalties for women

We further split the sample partly by region (urban or rural areas), income level, education level (longer or shorter schooling years), age (younger or older), the necessity of being a main caretaker of children (whether the mother was the primary caregiver), and reproductive age. The results are presented in Supplementary Fig. 3 and Supplementary Tables 13–18, suggesting that significant child penalties were observed for higher income younger mothers in urban areas who were caring for their children.

Given the existence in China of a preference for sons, we also examined the heterogeneity along the dimension of whether women gave birth to a son. Supplementary Fig. 4 and Table 19 show that over time, child penalties are more prevalent for those with a son. This is a bit surprising, but consistent with some literature showing that a daughter plays a larger role in taking care of parents compared to a son^25^. In addition to an extensive margin, we are also interested in how an intensive margin (e.g., the number of children conditional on at least one child) has an impact on mental health. However, this is not directly applicable in our strategy of event study. We thus show whether the results are heterogeneous between those with only one child and those with multiple children. Supplementary Fig. 5 and Table 20 indicates that child penalties are more prevalent for those with multiple children.

### Mediation analysis

To further explore the mechanisms through which childbirth affects women’s long-term health, we conducted a mediation model for further analysis. Following the literature^28,29^, we used three types of factors as mediators. The first type we adopted was economic/utilitarian factors including the receipt of financial support from parents and individual income. One of the reasons for women’s mental stress is that they feel financially constrained when raising a child. Literature shows that the rising child education expenditure triggered a large financial burden on families in China^30^. Most importantly, “tiger mothers” were more likely than fathers to focus on the educational investment on children^31^. If women perform well in the labor market with higher incomes or they receive more financial support from their parents, the anxiety of lack of money for raising children would be relieved. Second, we used psychological/emotional factors as mediators. We used co-residence with parents as one of the main proxies because most grandparents in China not only share the responsibility of raising grandchildren^25^, but also take care of women postpartum and provide them with emotional support. In addition, under the influence of Confucianism, having a child (especially a son) may lead to the improvement of women’s social status^32^, which may further influence women’s mental health. We thus further adopted women’s self-reported social status as another mediator. Third, we used self-reported physical health status as the proxy for physical factors. This variable is widely adopted as a control in mental health literature. However, in the case of childbearing, it may function as a mediator because physical injury caused by childbirth is very common and this may lead to mental health issues or even death.

Specifically, we recoded the event times into only two periods including period before childbirth (equal to 0) and period after childbirth (equal to 1). The results correspond to Eqs. (2) and (4) are reported in Supplementary Table 21, and the results corresponding to Eq. (3) are reported in Supplementary Table 22. It is found that (1) women’s mental health worsened after childbirth (−0.132, 95% CI: −0.219 to −0.045); (2) child birth leads to worsened physical health status (−0.037, 95% CI: −0.068 to −0.006) and lowered income (−0.439, 95% CI: −0.594 to −0.283); and (3) the coefficient of mental health remains significant but reduces with the inclusion of physical health status and income, falling to −0.124 (95% CI: −0.211 to −0.038) and −0.112 (95% CI: −0.202 to −0.022). Our results suggest that following childbirth women face physical health issues and earn less because of fewer labor market opportunities, which may lead to mental health issues. However, we do not find a mediating role for the other three variables including financial support from parents, co-residence with parents and self-reported social status.

## Discussion

Based on the data from the CFPS survey, our study used the event study method (around the birth of the first child) to examine the impact of childbirth on the mental health of men and women. It was found that although men and women had similar life satisfaction before parenthood, women experienced a significant, immediate, and long-term drop in life satisfaction and faced mental health issues after giving birth to the first child and even 10 years later, had not recovered. Statistically, men were found to be unaffected. Using significance tests, we further identified child penalties between men and women in the long (8-year) term. We also identified child penalties in labor market performance, measured by fewer opportunities, lower income and productivity, and physical health issues.

Despite a large body of literature focusing on the association between health and socio-economic status^33,34^, unemployment^35^, migration^36^, and intergenerational persistence^37^, only a few studies have focused on women’s mental health, although women are twice as likely as men to suffer from depression^38^. Even fewer studies have focused on how mothers’ mental health is linked to giving birth and raising a child. The few exceptions paying attention to women’s mental health status have mainly examined the impact over a very short period, mostly within 1 year postpartum. For instance, in an Australian context, one study reported that 16.1% of women reported depressive symptoms during the first 12 months postpartum;^16^ this was verified by another study which showed that 16.9% of women reported themselves as depressed 2 weeks postpartum^39^. Similar findings were identified in two European countries, France and Italy. In a survey conducted 12 months after childbirth, more than half the women reported anxiety, extreme tiredness, and backache, and one-third reported sleep disorders, depression, and other symptoms^40^. Some evidence in the US showed that work-family conflict was negatively associated with women’s mental health after childbirth (11 weeks postpartum)^41^.

In this study, we found that among female participants, rearing children had significant and persistent effects on mental health and subjective well-being. Men and women had an identical trend of mental health until the arrival of their first child; however, women’s life satisfaction score dropped by about 4.3% immediately after childbirth, while men did not seem to be affected, and this significant gap persisted for 8 years. These findings provide evidence to better understand the impact of the comparative advantage of the gender gap after childbirth in the context of mental health. Our study is in line with the aforementioned studies as they highlight the negative impact of childbirth on women’s mental health status. We further complemented the literature in two ways. First, we identified the impact over a longer period than a shorter one (e.g., 12 months postpartum) and considered the combined effects of childbirth on socioeconomic positions (e.g., labor market performance). Second, we examined the effects of childbirth on both women and men, and such comparisons suggest the existence of child penalties in mental health.

Previous studies have mainly focused on child penalties in the labor market. For instance, some studies have shown that the arrival of children creates a long-term gender gap of around 20% in earnings, driven by hours worked, participation, and wage rates^9^. This child penalty in earnings persists in different countries, such as Denmark, Austria, the US, and Germany^10^, and remains for biological and adoptive mothers^26^. Most importantly, child penalties are likely to increase gender inequality, which has been verified in the past few decades^9^. However, child penalties are not only reflected in the labor market performance, but also in the potentially negative impact on the health (especially mental health) of women (rather than men). This point has been overlooked in the literature but is highlighted in this study. We also advance the literature by showing that the negative impact of childbirth on women’s mental health is associated with child penalties in the labor market, suggesting that earning a lower income is one of the potential reasons for the worsening mental health of women compared to men. This study also adds to some of the evidence showing that mothers who had an unplanned cesarean section were at a higher risk of developing postnatal depression^42^ and that interventions aimed at improving birth-related outcomes have mental health spillovers^43^.

A limitation of this study was the absence of additional proxies for physical health status. We did not find any evidence of child penalties in physical health status proxied by an indicator of chronic disease in this study. Specifically, neither women nor men were likely to have a higher probability of developing chronic diseases after childbirth. This finding contradicts some studies suggesting that childbirth worsens physical health for women^17,44,45^, which is also associated with mental health^16^. The indicators for physical health in these studies included short-term back pain, breast problems, bowel problems, and painful perineum, while the proxy used in this study—chronic disease—was more likely to occur over a longer period. When employing two other measures for physical health status, we found that there was a higher probability of hospitalization for women in the first two years postpartum and throughout the 10 years after childbirth there was a lower probability of reporting improved physical health. Thus, we interpreted this finding with caution, highlighting that child penalties in mental health were also likely to be associated with the negative impacts of childbirth on physical health status.

Another limitation was that we did not show whether and how child penalties in mental health have changed over the past few decades in China. Due to data limitations, we were unable to capture this trend, which would have great policy implications for China to achieve a society with gender equity. Additionally, it was beyond the scope of this research to compare China to other developing and developed countries. Fundamentally, a global comparison would help us advance toward SDG3 and SDG5.

Notwithstanding the limitations, our study contributes to the literature on child penalties. Much of the literature has focused on the impact of labor market outcomes, but little attention has been paid to mental health. Drawing on related child penalty studies, we find that child penalties in mental health are also substantial, which is likely associated with child penalties in labor market performance. This is marked by fewer opportunities and lower income and physical health for women. The study’s findings suggest that to achieve SDG3 and SDG5, more attention should be directed to women’s mental health since they are shown to suffer more from childbirth in comparison to men. A supportive healthcare system could be a feasible solution to aid women in coping with mental health issues. While the healthcare system often neglects women’s postpartum concerns, facilitating family and group support for women is also necessary. While the macro-level association between demographic transition and economic growth has been studied in detail, few studies have focused on the micro-level implications for women. When countries that face the problem of the so-called “low-fertility trap” attempt to adopt multiple tools to stimulate the birth rate for economic growth, they must simultaneously consider the implicit burden on women. Concerted action to ensure equal rights in the labor market after childbirth is needed at all levels—from governments to enterprises, individuals, civil society, and global initiatives. A mixed tool of the supportive healthcare system and labor market policies (e.g., eliminating gender discrimination) has increasingly become one of the central tasks to help women facing child penalties. Further research is needed on adopting cross-country data covering a longer period, allowing examination of the trends in child penalties in China and other countries.

### Supplementary information

Reporting Summary

Supplementary Information

## Data Availability

The data used in this study are available at http://www.isss.pku.edu.cn/cfps/en/index.htm.

## Code availability

The codes are available upon reasonable request.
