# Supplementary material for: Mental health penalties of having a child: findings from the China family panel studies
Source: Npj Ment Health Res. 2023 May 15;2:7. doi: 10.1038/s44184-023-00026-x (PMC10184102; doi:10.1038/s44184-023-00026-x)
Supplement: Supplementary file 2 — Supplementary Information [file 44184_2023_26_MOESM2_ESM.pdf]

Supplementary Information

Mental Health Penalties of Having a Child: Findings from the China Family Panel Studies

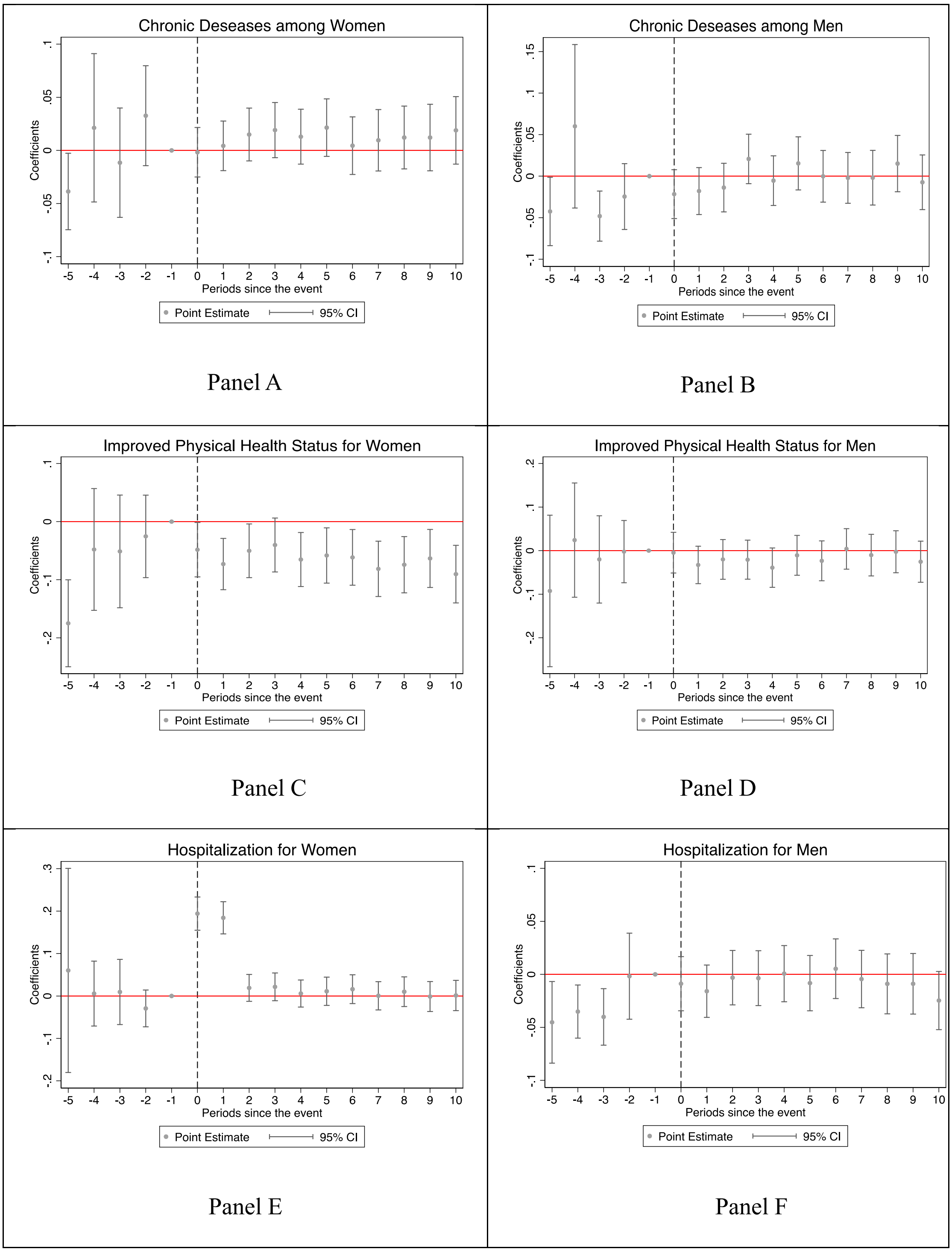

Supplementary Figure 1. Child penalties in physical health

Each dot represents a regression coefficient and the corresponding vertical line (error bar) represents the 95% confidence interval. Schooling years, age-fixed effects, city-fixed effects, year-fixed effects, province-year-fixed effects, the year-of-survey-fixed effects, and the month-of-survey-fixed effects are included. When the 0 from y axis is not covered by the vertical line, it signifies that the coefficient is significant and different from zero. The dashed gray line in event 0 signifies the year of arrival of the first child.

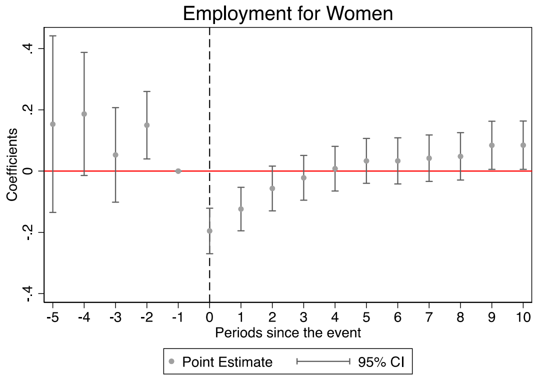

Panel A

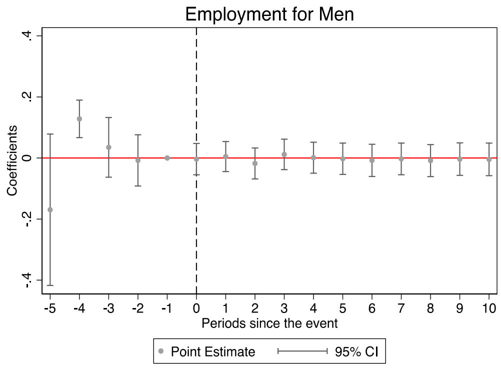

Panel B

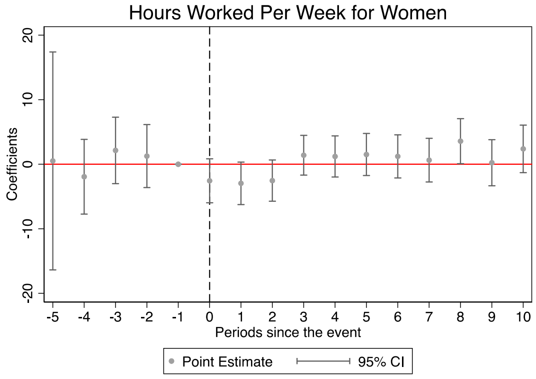

Panel C

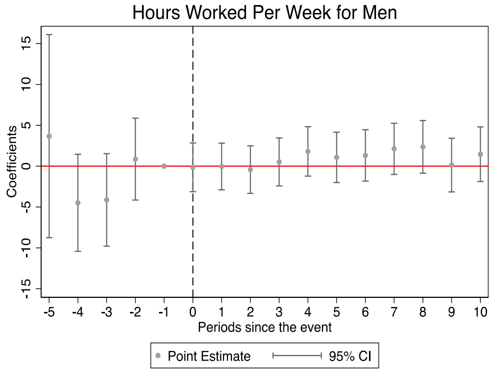

Panel D

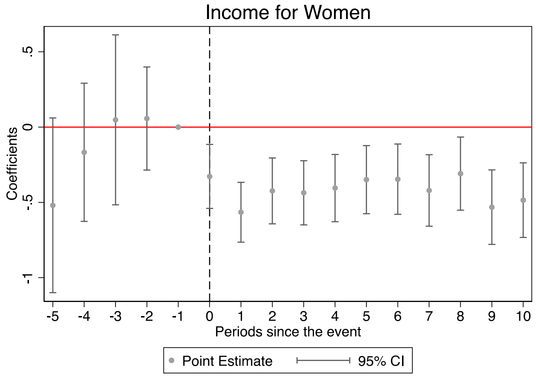

Panel E

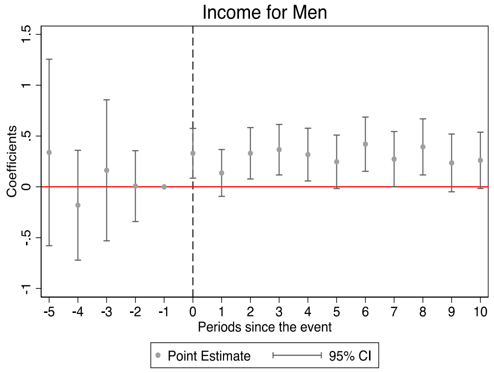

Panel F

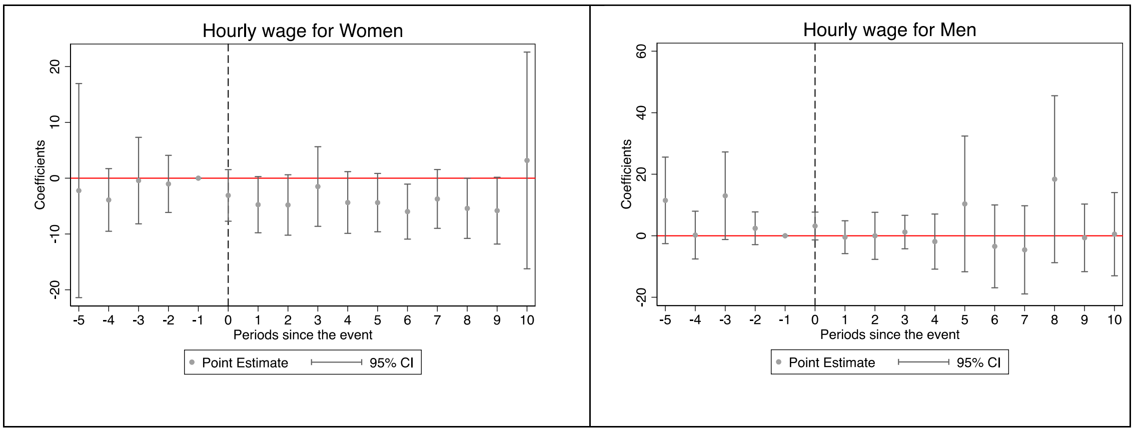

Panel G Panel H

Supplementary Figure 2. Child penalties in labor market performance

Each dot represents a regression coefficient and the corresponding vertical line (error bar) represents the 95% confidence interval. Schooling years, age-fixed effects, city-fixed effects, year-fixed effects, province-year-fixed effects, the year-of-survey-fixed effects, and the month-of-survey-fixed effects are included. When the 0 from y axis is not covered by the vertical line, it signifies that the coefficient is significant and different from zero. The dashed gray line in event 0 signifies the year of arrival of the first child.

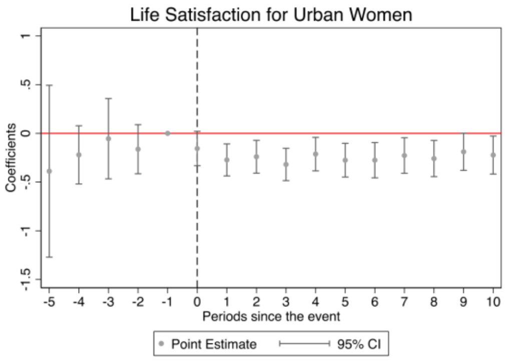

Panel A

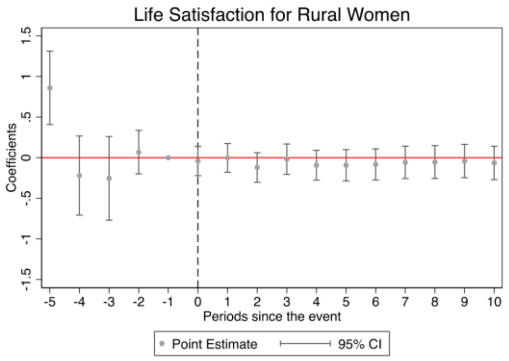

Panel B

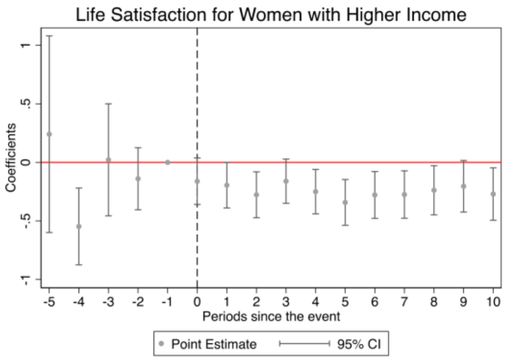

Panel C

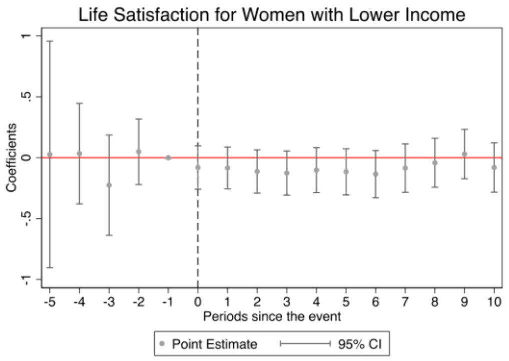

Panel D

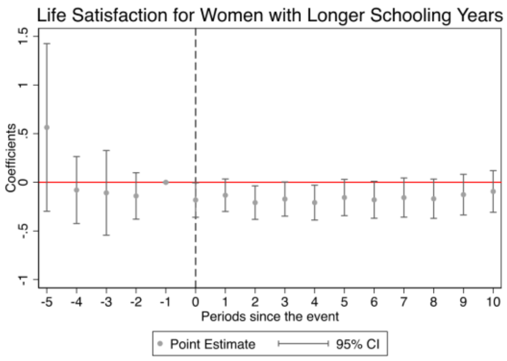

Panel E

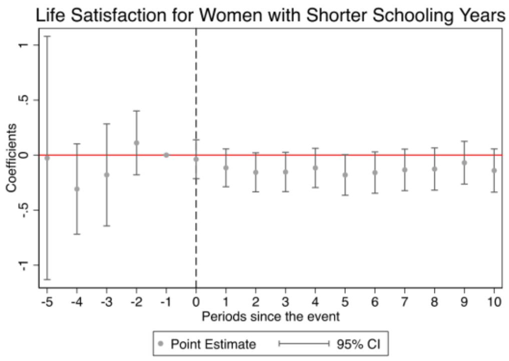

Panel F

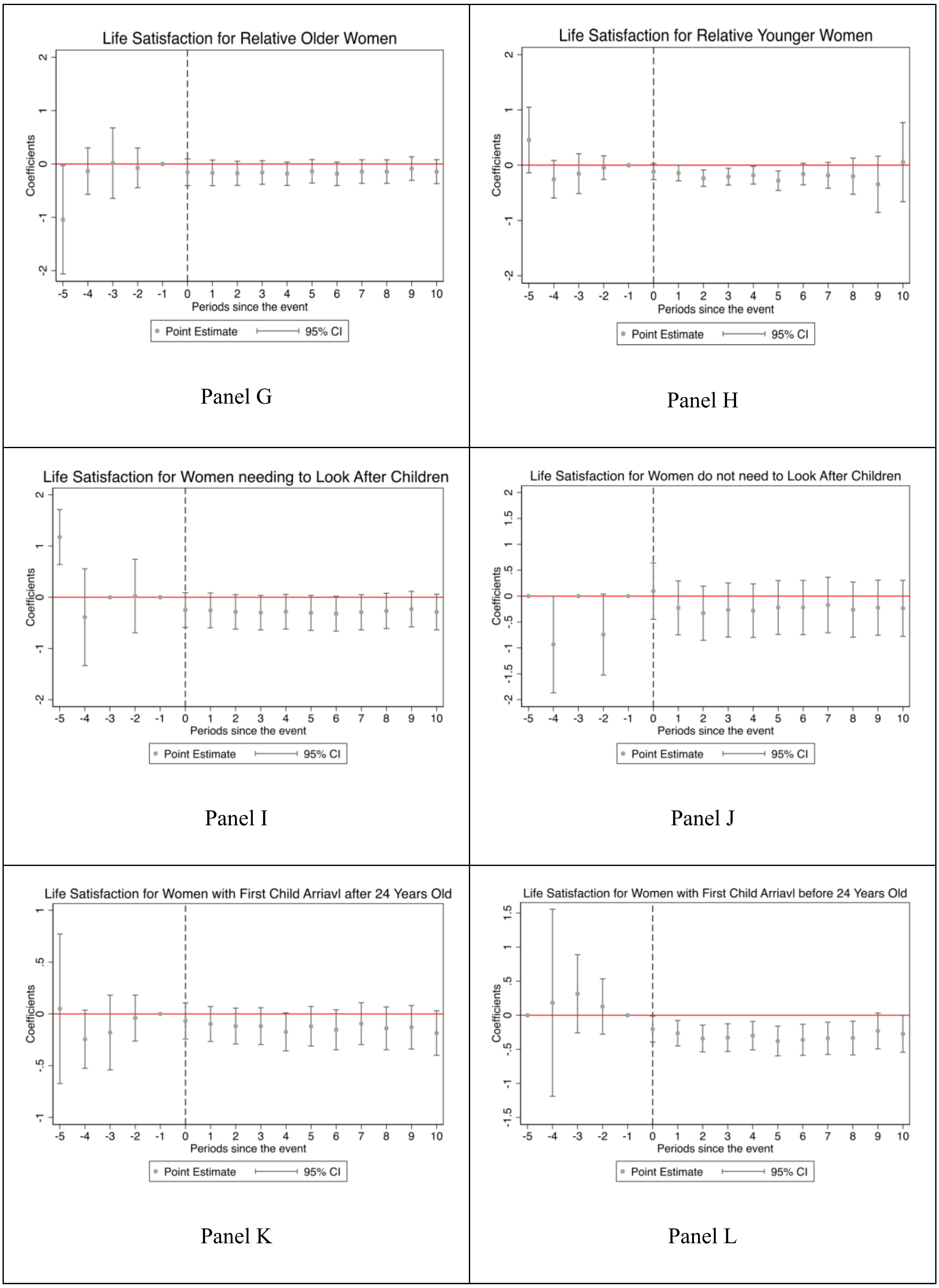

Supplementary Figure 3. Heterogeneity analysis

Each dot represents a regression coefficient, and the corresponding vertical line (error bar) represents the 95% confidence interval. Schooling years, age-fixed effects, city-fixed effects, year-fixed effects, province-year-fixed effects, the year-of-survey-fixed effects, and the month-of-survey-fixed effects are included. When the 0 from y axis is not covered by the vertical line, it signifies that the coefficient is significant and different from zero. The dashed gray line in event 0 signifies the year of arrival of the first child.

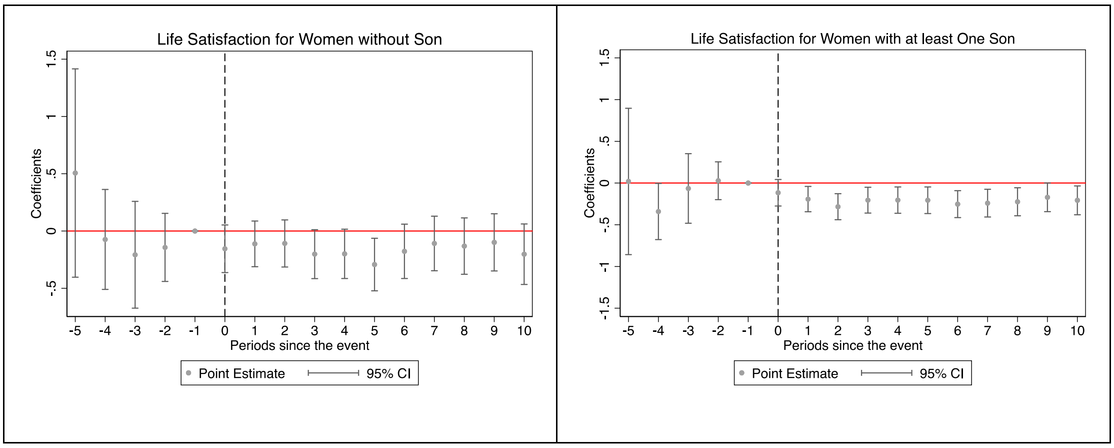

Panel A Panel B

Supplementary Figure 4. Life satisfaction for women with and without a son

Each dot represents a regression coefficient and the corresponding vertical line (error bar) represents the 95% confidence interval. Schooling years, age-fixed effects, city-fixed effects, year-fixed effects, province-year-fixed effects, the year-of-survey-fixed effects, and the month-of-survey-fixed effects are included. When the 0 from y axis is not covered by the vertical line, it signifies that the coefficient is significant and different from zero. The dashed gray line in event 0 signifies the year of arrival of the first child.

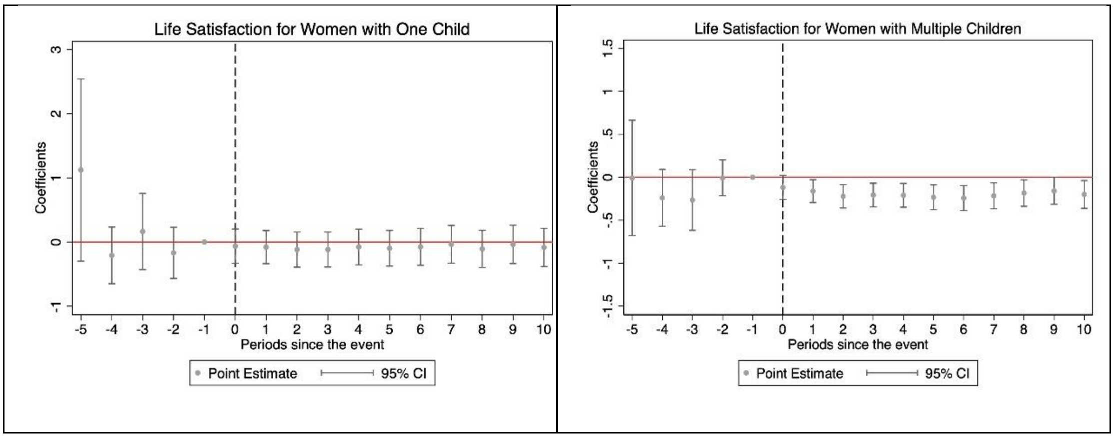

Panel A Panel B

Supplementary Figure 5. Life satisfaction for women with one child and with multiple children

Each dot represents a regression coefficient and the corresponding vertical line (error bar) represents the 95% confidence interval. Schooling years, age-fixed effects, city-fixed effects, year-fixed effects, province-year-fixed effects, the yea-of-survey-fixed-effects, and the month-of-survey-fixed effects are included. When the 0 from y axis is not covered by the vertical line, it signifies that the coefficient is significant and different from zero. The dashed gray line in event 0 signifies the year of arrival of the first child.

Supplementary Table 1. Impact of childbirth on life satisfaction for women with additional controls

	(1)	(2)	(3)
		Life satisfaction	
u_b5	0.125	0.101	
	[-0.539,0.789]	[-0.565,0.767]	
u_b4	-0.214	-0.231*	-0.518
	[-0.478,0.051]	[-0.496,0.034]	[-1.174,0.137]
u_b3	-0.101	-0.117	0.415
	[-0.418,0.215]	[-0.435,0.201]	[-0.245,1.075]
u_b2	-0.051	-0.055	0.057
	[-0.233,0.131]	[-0.236,0.127]	[-0.274,0.388]
u_a0	-0.107*	-0.103*	-0.164
	[-0.230,0.017]	[-0.226,0.020]	[-0.386,0.058]
u_a1	-0.146**	-0.141**	-0.160
	[-0.264,-0.029]	[-0.259,-0.023]	[-0.371,0.052]
u_a2	-0.198***	-0.185***	-0.244**
	[-0.319,-0.077]	[-0.308,-0.063]	[-0.464,-0.025]
u_a3	-0.178***	-0.164***	-0.190*
	[-0.299,-0.056]	[-0.287,-0.040]	[-0.414,0.034]
u_a4	-0.172***	-0.158**	-0.233**
	[-0.296,-0.049]	[-0.284,-0.031]	[-0.461,-0.006]
u_a5	-0.190***	-0.176***	-0.248**
	[-0.317,-0.063]	[-0.307,-0.044]	[-0.483,-0.013]
u_a6	-0.194***	-0.170**	-0.248**
	[-0.324,-0.065]	[-0.305,-0.035]	[-0.487,-0.008]
u_a7	-0.170**	-0.140*	-0.269**
	[-0.302,-0.037]	[-0.280,0.001]	[-0.514,-0.024]
u_a8	-0.170**	-0.139*	-0.236*
	[-0.304,-0.035]	[-0.282,0.005]	[-0.489,0.018]
u_a9	-0.114	-0.083	-0.194
	[-0.252,0.024]	[-0.232,0.065]	[-0.455,0.067]
u_a10	-0.169**	-0.129*	-0.232*
	[-0.308,-0.029]	[-0.283,0.024]	[-0.502,0.037]
Further controls	Y	Y	Y
N	11,449	11,520	5,809
R^2	0.097	0.097	0.137

This table shows the results of event study for women. The dependent variable is life satisfaction. The independent variables are event time dummies that are identical to those in Table 2. In additional to the controls in Table 2, column 1 further controls for the number of children and the number of sons, column 2 further controls for whether they have borne at least two children and their age when the last child was born. In column 3, we restrict the sample to those who have borne at least two children and control for their age when the second child was born. 95% confidence intervals are in brackets, with *p < 0.1, **p < 0.05, and ***p < 0.01.

Supplementary Table 2. Impact of childbirth on life satisfaction for men with additional controls

	(1)	(2)	(3)
		Life satisfaction	
u_b5	0.274	0.257	
	[-0.365,0.914]	[-0.384,0.897]	
u_b4	-0.146	-0.156	-0.390*
	[-0.430,0.137]	[-0.441,0.128]	[-0.797,0.017]
u_b3	0.210	0.201	-0.252
	[-0.148,0.568]	[-0.159,0.561]	[-0.972,0.469]
u_b2	0.080	0.077	-0.119
	[-0.122,0.281]	[-0.125,0.279]	[-0.481,0.242]
u_a0	0.061	0.068	0.148
	[-0.078,0.199]	[-0.070,0.206]	[-0.099,0.395]
u_a1	0.018	0.014	0.030
	[-0.108,0.145]	[-0.113,0.142]	[-0.204,0.264]
u_a2	0.028	0.037	0.061
	[-0.107,0.163]	[-0.099,0.174]	[-0.181,0.303]
u_a3	-0.005	0.005	0.028
	[-0.135,0.126]	[-0.127,0.138]	[-0.210,0.267]
u_a4	0.031	0.047	0.034
	[-0.107,0.169]	[-0.095,0.189]	[-0.221,0.289]
u_a5	0.062	0.074	0.061
	[-0.077,0.200]	[-0.069,0.217]	[-0.194,0.315]
u_a6	0.039	0.057	0.085
	[-0.102,0.181]	[-0.092,0.205]	[-0.178,0.347]
u_a7	0.039	0.059	0.118
	[-0.105,0.182]	[-0.093,0.211]	[-0.147,0.383]
u_a8	0.135*	0.157**	0.164
	[-0.011,0.281]	[0.001,0.313]	[-0.110,0.438]
u_a9	0.053	0.078	0.089
	[-0.097,0.203]	[-0.083,0.239]	[-0.193,0.371]
u_a10	0.073	0.102	0.121
	[-0.078,0.224]	[-0.063,0.268]	[-0.171,0.413]
Control	Y	Y	Y
N	10,416	10,495	5,182
R^2	0.117	0.117	0.147

This table shows the results of event study for men. The dependent variable is life satisfaction. The independent variables are event time dummies that are identical to those in Table 2. In additional to the controls in Table 2, column 1 further controls for the number of children and the number of sons, column 2 further controls for whether they have borne at least two children and their age when the last child was born. In column 3, we restrict the sample to those who have borne at least two children and control for their age when the second child was born. 95% confidence intervals are in brackets, with *p < 0.1, **p < 0.05, and ***p < 0.01.

Supplementary Table 3. Impact of childbirth on life satisfaction considering unbalanced panel issues

	(1)	(2)	(3)	(4)
	Surveyed more than once		Had the first child after 2010	
	Female	Male	Female	Male
u_b5	0.108	0.210	0.105	0.297
	[-0.541,0.758]	[-0.455,0.875]	[-0.565,0.775]	[-0.385,0.980]
u_b4	-0.175	-0.043	-0.151	-0.139
	[-0.444,0.093]	[-0.337,0.250]	[-0.431,0.128]	[-0.442,0.163]
u_b3	-0.142	0.285	-0.128	0.244
	[-0.491,0.207]	[-0.111,0.681]	[-0.449,0.194]	[-0.106,0.595]
u_b2	-0.066	0.088	-0.053	0.074
	[-0.263,0.131]	[-0.132,0.308]	[-0.238,0.133]	[-0.128,0.275]
u_a0	-0.114*	0.061	-0.124*	0.056
	[-0.248,0.021]	[-0.091,0.213]	[-0.250,0.002]	[-0.086,0.197]
u_a1	-0.116*	-0.002	-0.149**	-0.041
	[-0.241,0.010]	[-0.138,0.134]	[-0.282,-0.015]	[-0.181,0.100]
u_a2	-0.215***	-0.022	-0.232***	-0.002
	[-0.345,-0.086]	[-0.168,0.124]	[-0.368,-0.095]	[-0.153,0.150]
u_a3	-0.163**	-0.042	-0.200***	-0.071
	[-0.292,-0.033]	[-0.183,0.099]	[-0.349,-0.051]	[-0.227,0.084]
u_a4	-0.189***	-0.011	-0.171**	-0.013
	[-0.322,-0.057]	[-0.161,0.139]	[-0.319,-0.023]	[-0.180,0.154]
u_a5	-0.205***	0.012	-0.220**	-0.065
	[-0.341,-0.069]	[-0.137,0.161]	[-0.393,-0.046]	[-0.251,0.121]
u_a6	-0.196***	0.010	-0.201**	-0.015
	[-0.335,-0.058]	[-0.143,0.164]	[-0.376,-0.025]	[-0.210,0.181]
u_a7	-0.157**	0.029	-0.223**	-0.096
	[-0.299,-0.015]	[-0.126,0.184]	[-0.417,-0.029]	[-0.315,0.124]
u_a8	-0.166**	0.108	-0.153	0.087
	[-0.311,-0.022]	[-0.049,0.266]	[-0.367,0.060]	[-0.144,0.319]
u_a9	-0.140*	-0.029		
	[-0.294,0.014]	[-0.198,0.139]		
u_a10	-0.152*	0.069		
	[-0.308,0.005]	[-0.101,0.239]		
Control	Y	Y	Y	Y
N	9,591	8,596	5,174	4,728
R^2	0.101	0.127	0.121	0.144

This table shows the results of event study for women and men, respectively. The dependent variable is life satisfaction. In columns (1) and (2), we excluded those who were only surveyed once in 5 waves. In columns (3) and (4), we only include those who gave birth to the first child after 2010. The independent variables are event time dummies, indexed such that u_bt means t years before the year of arrival of first child, and u_at means t years after the year of arrival of the first child, respectively. The event-time dummy at t = -1 was omitted, implying that the event-time coefficients measure the impact of children relative to the year just before the first childbirth. Individual controls included years of schooling and age fixed effects to control for life cycle trends. City-fixed effects, year-fixed effects, province-year-fixed effects, the year-of-survey-fixed effects, and the month-of-survey-fixed effects were included. 95% confidence intervals are in brackets, with *p < 0.1, **p < 0.05, and ***p < 0.01.

Supplementary Table 4. Impact of childbirth on depression for women and men

Depression	(1)<br>Female	(2)<br>Male
u_b5	0.258	0.033
	[-0.341,0.858]	[-0.351,0.416]
u_b4	0.333***	-0.040
	[0.097,0.569]	[-0.240,0.159]
u_b3	-0.048	-0.122
	[-0.216,0.120]	[-0.280,0.037]
u_b2	0.119*	0.060
	[-0.019,0.256]	[-0.068,0.189]
u_a0	0.056	-0.012
	[-0.028,0.140]	[-0.088,0.064]
u_a1	0.103**	0.039
	[0.022,0.185]	[-0.035,0.114]
u_a2	0.106**	0.011
	[0.023,0.189]	[-0.067,0.089]
u_a3	0.122***	0.039
	[0.038,0.206]	[-0.038,0.117]
u_a4	0.118***	0.023
	[0.034,0.202]	[-0.056,0.101]
u_a5	0.117***	0.029
	[0.031,0.202]	[-0.050,0.108]
u_a6	0.137***	-0.004
	[0.046,0.228]	[-0.087,0.078]
u_a7	0.091**	-0.007
	[0.001,0.181]	[-0.090,0.076]
u_a8	0.127***	-0.016
	[0.033,0.220]	[-0.101,0.070]
u_a9	0.140***	-0.027
	[0.044,0.235]	[-0.114,0.060]
u_a10	0.103**	-0.036
	[0.007,0.199]	[-0.125,0.053]
Control	Y	Y
N	11,504	10,482
R^2	0.055	0.070

This table shows the results of event study with the dependent variable being depression. The independent variables are event time dummies, indexed such that u_bt means t years before the year of arrival of first child, and u_at means t years after the year of arrival of the first child, respectively. The event-time dummy at t = -1 was omitted, implying that the event-time coefficients measure the impact of children relative to the year just before the first childbirth. Individual controls included years of schooling and age fixed effects to control for life cycle trends. City-fixed effects, year-fixed effects, province-year-fixed effects, the year-of-survey-fixed effects, and the month-of-survey-fixed effects were included. 95% confidence intervals are in brackets, with *p < 0.1, **p < 0.05, and ***p < 0.01.

Supplementary Table 5. Impact of childbirth on life satisfaction and depression for women and men (weights adjusted)

	(1)	(2)	(3)	(4)
	Women		Men	
	Life satisfaction	Depression	Life satisfaction	Depression
u_b5	-0.242	-0.244	0.254	0.219
	[-1.201,0.716]	[-0.679,0.191]	[-0.317,0.824]	[-0.154,0.593]
u_b4	-0.223	0.482**	-0.122	-0.129
	[-0.554,0.107]	[0.075,0.889]	[-0.476,0.233]	[-0.357,0.098]
u_b3	0.020	-0.080	0.294	-0.130
	[-0.391,0.430]	[-0.298,0.139]	[-0.105,0.693]	[-0.303,0.042]
u_b2	0.031	0.128	0.071	0.035
	[-0.222,0.284]	[-0.043,0.300]	[-0.169,0.311]	[-0.122,0.191]
u_a0	-0.041	0.084	0.079	-0.014
	[-0.213,0.131]	[-0.035,0.202]	[-0.087,0.245]	[-0.108,0.080]
u_a1	-0.069	0.087	0.064	0.015
	[-0.226,0.088]	[-0.024,0.197]	[-0.091,0.218]	[-0.078,0.107]
u_a2	-0.064	0.079	0.017	-0.041
	[-0.227,0.099]	[-0.033,0.191]	[-0.146,0.179]	[-0.135,0.053]
u_a3	-0.163**	0.087	0.003	-0.019
	[-0.325,-0.002]	[-0.028,0.203]	[-0.159,0.164]	[-0.114,0.075]
u_a4	-0.112	0.120**	0.039	-0.006
	[-0.277,0.052]	[0.007,0.232]	[-0.127,0.205]	[-0.101,0.089]
u_a5	-0.148*	0.116**	0.080	-0.023
	[-0.315,0.020]	[0.003,0.229]	[-0.086,0.247]	[-0.116,0.071]
u_a6	-0.127	0.166***	0.029	-0.022
	[-0.295,0.040]	[0.045,0.287]	[-0.141,0.199]	[-0.121,0.077]
u_a7	-0.113	0.095	0.074	-0.024
	[-0.284,0.058]	[-0.023,0.213]	[-0.096,0.244]	[-0.126,0.077]
u_a8	-0.114	0.126**	0.119	-0.052
	[-0.286,0.059]	[0.006,0.246]	[-0.054,0.292]	[-0.156,0.052]
u_a9	-0.043	0.159**	0.052	-0.072
	[-0.217,0.132]	[0.035,0.282]	[-0.126,0.230]	[-0.177,0.033]
u_a10	-0.125	0.112*	0.056	-0.079
	[-0.304,0.054]	[-0.011,0.235]	[-0.123,0.235]	[-0.186,0.028]
Control	Y	Y	Y	Y
N	8,932	8,921	8,992	8,981
R^2	0.103	0.067	0.117	0.076

This table shows the results of event study with sampling weights adjusted for women (columns 1-2) and men (columns 3-4) respectively. The dependent variables include life satisfaction and depression. The independent variables are event time dummies, indexed such that u_bt means t years before the year of arrival of first child, and u_at means t years after the year of arrival of the first child, respectively. The event-time dummy at t = -1 was omitted, implying that the event-time coefficients measure the impact of children relative to the year just before the first childbirth. Individual controls included years of schooling and age fixed effects to control for life cycle trends. City-fixed effects, year-fixed effects, province-year-fixed effects, the year-of-survey-fixed effects, and the-month-of-survey-fixed effects were included. 95% confidence intervals are in brackets, with *p < 0.1, **p < 0.05, and ***p < 0.01.

Supplementary Table 6. Impact of childbirth on chronic disease for women and men

	(1)	(2)	(3)	(4)
	Having chronic disease			
	Women	Men	Significance of the difference	
u_b5	-0.039**	-0.043**	u_b5_chi2	0.021
	[-0.075,-0.003]	[-0.084,-0.001]	u_b5_p	0.885
u_b4	0.021	0.060	u_b4_chi2	0.408
	[-0.049,0.091]	[-0.038,0.158]	u_b4_p	0.523
u_b3	-0.012	-0.048***	u_b3_chi2	1.475
	[-0.063,0.040]	[-0.078,-0.018]	u_b3_p	0.225
u_b2	0.033	-0.025	u_b2_chi2	3.436
	[-0.014,0.080]	[-0.064,0.015]	u_b2_p	0.064
u_a0	-0.002	-0.022	u_a0_chi2	1.125
	[-0.025,0.022]	[-0.051,0.008]	u_a0_p	0.289
u_a1	0.004	-0.018	u_a1_chi2	1.409
	[-0.019,0.028]	[-0.046,0.010]	u_a1_p	0.235
u_a2	0.015	-0.014	u_a2_chi2	2.262
	[-0.010,0.040]	[-0.043,0.015]	u_a2_p	0.133
u_a3	0.019	0.021	u_a3_chi2	0.007
	[-0.007,0.045]	[-0.009,0.051]	u_a3_p	0.935
u_a4	0.013	-0.005	u_a4_chi2	0.864
	[-0.013,0.039]	[-0.035,0.025]	u_a4_p	0.352
u_a5	0.021	0.015	u_a5_chi2	0.085
	[-0.006,0.048]	[-0.017,0.047]	u_a5_p	0.770
u_a6	0.004	-0.000	u_a6_chi2	0.050
	[-0.023,0.032]	[-0.031,0.031]	u_a6_p	0.823
u_a7	0.010	-0.002	u_a7_chi2	0.303
	[-0.019,0.038]	[-0.033,0.029]	u_a7_p	0.582
u_a8	0.012	-0.002	u_a8_chi2	0.411
	[-0.017,0.042]	[-0.035,0.031]	u_a8_p	0.521
u_a9	0.012	0.015	u_a9_chi2	0.018
	[-0.019,0.043]	[-0.019,0.049]	u_a9_p	0.893
u_a10	0.019	-0.007	u_a10_chi2	1.356
	[-0.013,0.051]	[-0.040,0.026]	u_a10_p	0.244
Control	Y	Y		
N	11,518	10,492		
R-sq	0.040	0.050		

Columns (1) and (2) show the impact of having a child on chronic disease for men and women, respectively. The independent variables are event time dummies, indexed such that u_bt means t years before the year of arrival of first child, and u_at means t years after the year of arrival of the first child, respectively. The event-time dummy at t = -1 is omitted, implying that the event-time coefficients measure the impact of children relative to the year just before the first childbirth. Individual controls included years of schooling and age fixed effects to control for life cycle trends. City-fixed effects, year-fixed effects, province-year-fixed effects, the year-of-survey-fixed effects, and the month-of-survey-fixed effects are included. To test whether the coefficients by subgroups are significantly different, we use the test drawing on seemingly unrelated regressions with the command suest in Stata. *p < 0.1, **p < 0.05, and ***p < 0.01, 95% confidence intervals are in brackets.

Supplementary Table 7. Impact of childbirth on self-reported physical health status for women and men

	(1)	(2)	(3)	(4)
	Improved physical health status			
	Women	Men	Significance of the difference	
u_b5	-0.175***	-0.093	u_b5_chi2	0.747
	[-0.250,-0.100]	[-0.267,0.081]	u_b5_p	0.387
u_b4	-0.048	0.024	u_b4_chi2	0.727
	[-0.153,0.057]	[-0.107,0.155]	u_b4_p	0.394
u_b3	-0.051	-0.020	u_b3_chi2	0.192
	[-0.148,0.046]	[-0.120,0.080]	u_b3_p	0.661
u_b2	-0.025	-0.002	u_b2_chi2	0.206
	[-0.096,0.046]	[-0.074,0.069]	u_b2_p	0.650
u_a0	-0.048**	-0.005	u_a0_chi2	1.720
	[-0.095,-0.002]	[-0.051,0.042]	u_a0_p	0.190
u_a1	-0.073***	-0.033	u_a1_chi2	1.552
	[-0.117,-0.029]	[-0.076,0.010]	u_a1_p	0.213
u_a2	-0.050**	-0.020	u_a2_chi2	0.854
	[-0.096,-0.004]	[-0.066,0.025]	u_a2_p	0.355
u_a3	-0.040*	-0.021	u_a3_chi2	0.359
	[-0.087,0.006]	[-0.066,0.024]	u_a3_p	0.549
u_a4	-0.065***	-0.039*	u_a4_chi2	0.657
	[-0.112,-0.019]	[-0.084,0.006]	u_a4_p	0.418
u_a5	-0.058**	-0.011	u_a5_chi2	2.114
	[-0.106,-0.011]	[-0.056,0.035]	u_a5_p	0.146
u_a6	-0.062**	-0.023	u_a6_chi2	1.353
	[-0.109,-0.014]	[-0.069,0.022]	u_a6_p	0.245
u_a7	-0.081***	0.004	u_a7_chi2	6.615
	[-0.129,-0.034]	[-0.043,0.050]	u_a7_p	0.010
u_a8	-0.074***	-0.010	u_a8_chi2	3.639
	[-0.123,-0.026]	[-0.058,0.038]	u_a8_p	0.056
u_a9	-0.063**	-0.003	u_a9_chi2	3.189
	[-0.113,-0.013]	[-0.051,0.046]	u_a9_p	0.074
u_a10	-0.090***	-0.025	u_a10_chi2	3.728
	[-0.140,-0.041]	[-0.073,0.022]	u_a10_p	0.054
Control	Y	Y		
N	11,729	10,865		
R-sq	0.047	0.054		

Columns (1) and (2) show the impact of having a child on self-reported physical health status (improved health status is equal to 1) for men and women, respectively. The independent variables are event time dummies, indexed such that u_bt means t years before the year of arrival of first child, and u_at means t years after the year of arrival of the first child, respectively. The event-time dummy at $t = -1$ is omitted, implying that the event-time coefficients measure the impact of children relative to the year just before the first childbirth. Individual controls included years of schooling and age fixed effects to control for life cycle trends. City-fixed effects, year-fixed effects, province-year-fixed effects, the year-of-survey-fixed effects, and the month-of-survey-fixed effects are included. To test whether the coefficients by subgroups are significantly different, we use the test drawing on seemingly unrelated regressions with the command suest in Stata. *p < 0.1, **p < 0.05, and ***p < 0.01, 95% confidence intervals are in brackets.

Supplementary Table 8. Impact of childbirth on hospitalization for women and men

	(1)	(2)	(3)	(4)
	Hospitalization			
	Women	Men	Significance of the difference	
u_b5	0.060	-0.045**	u_b5_chi2	0.743
	[-0.180,0.301]	[-0.084,-0.007]	u_b5_p	0.389
u_b4	0.006	-0.035***	u_b4_chi2	1.024
	[-0.071,0.082]	[-0.060,-0.010]	u_b4_p	0.312
u_b3	0.009	-0.040***	u_b3_chi2	1.510
	[-0.067,0.086]	[-0.067,-0.013]	u_b3_p	0.219
u_b2	-0.029	-0.002	u_b2_chi2	0.856
	[-0.073,0.014]	[-0.042,0.039]	u_b2_p	0.355
u_a0	0.194***	-0.009	u_a0_chi2	75.127
	[0.155,0.233]	[-0.034,0.017]	u_a0_p	0.000
u_a1	0.184***	-0.016	u_a1_chi2	79.119
	[0.146,0.222]	[-0.041,0.009]	u_a1_p	0.000
u_a2	0.019	-0.003	u_a2_chi2	1.172
	[-0.013,0.051]	[-0.029,0.023]	u_a2_p	0.279
u_a3	0.021	-0.004	u_a3_chi2	1.474
	[-0.011,0.054]	[-0.030,0.022]	u_a3_p	0.225
u_a4	0.006	0.001	u_a4_chi2	0.061
	[-0.026,0.038]	[-0.026,0.027]	u_a4_p	0.805
u_a5	0.011	-0.008	u_a5_chi2	0.851
	[-0.022,0.044]	[-0.034,0.018]	u_a5_p	0.356
u_a6	0.016	0.005	u_a6_chi2	0.238
	[-0.018,0.050]	[-0.023,0.033]	u_a6_p	0.626
u_a7	0.001	-0.004	u_a7_chi2	0.053
	[-0.033,0.034]	[-0.032,0.023]	u_a7_p	0.818
u_a8	0.010	-0.009	u_a8_chi2	0.718
	[-0.025,0.045]	[-0.037,0.019]	u_a8_p	0.397
u_a9	-0.001	-0.009	u_a9_chi2	0.112
	[-0.037,0.034]	[-0.037,0.020]	u_a9_p	0.738
u_a10	0.001	-0.025*	u_a10_chi2	1.315
	[-0.034,0.037]	[-0.052,0.003]	u_a10_p	0.251
Control	Y	Y		
N	11,519	10,494		
R-sq	0.088	0.045		

Columns (1) and (2) show the impact of having a child on hospitalization (equals to 1 if hospitalized last year) for men and women, respectively. The independent variables are event time dummies, indexed such that u_bt mean t years before the year of arrival of first child, and u_at means t years after the year of arrival of the first child, respectively. The event-time dummy at t = -1 is omitted, implying that the event-time coefficients measure the impact of children relative to the year just before the first childbirth. Individual controls included years of schooling and age fixed effects to control for life cycle trends. City-fixed effects, year-fixed effects, province-year-fixed effects, the year-of-survey-fixed effects, and the month-of-survey-fixed effects are included. To test whether the coefficients by subgroups are significantly different, we use the test drawing on seemingly unrelated regressions with the command suest in Stata. *p < 0.1, **p < 0.05, and ***p < 0.01, 95% confidence intervals are in brackets.

Supplementary Table 9. Impact of childbirth on employment for women and men

	(1)	(2)	(3)	(4)
	Whether working			
	Women	Men	Significance of the difference	
u_b5	0.153	-0.169	u_b5_chi2	2.892
	[-0.135,0.441]	[-0.417,0.078]	u_b5_p	0.089
u_b4	0.186*	0.128***	u_b4_chi2	0.309
	[-0.014,0.387]	[0.067,0.190]	u_b4_p	0.578
u_b3	0.053	0.035	u_b3_chi2	0.034
	[-0.102,0.207]	[-0.063,0.133]	u_b3_p	0.853
u_b2	0.150***	-0.008	u_b2_chi2	5.346
	[0.040,0.260]	[-0.092,0.076]	u_b2_p	0.021
u_a0	-0.195***	-0.004	u_a0_chi2	18.119
	[-0.270,-0.121]	[-0.055,0.048]	u_a0_p	0.000
u_a1	-0.124***	0.005	u_a1_chi2	8.338
	[-0.195,-0.053]	[-0.044,0.054]	u_a1_p	0.004
u_a2	-0.057	-0.018	u_a2_chi2	0.775
	[-0.130,0.016]	[-0.068,0.033]	u_a2_p	0.379
u_a3	-0.022	0.012	u_a3_chi2	0.585
	[-0.095,0.051]	[-0.038,0.062]	u_a3_p	0.444
u_a4	0.008	0.001	u_a4_chi2	0.025
	[-0.065,0.081]	[-0.050,0.052]	u_a4_p	0.875
u_a5	0.033	-0.003	u_a5_chi2	0.657
	[-0.040,0.106]	[-0.054,0.049]	u_a5_p	0.418
u_a6	0.033	-0.008	u_a6_chi2	0.841
	[-0.042,0.109]	[-0.061,0.045]	u_a6_p	0.359
u_a7	0.042	-0.003	u_a7_chi2	0.995
	[-0.034,0.118]	[-0.055,0.049]	u_a7_p	0.318
u_a8	0.048	-0.008	u_a8_chi2	1.527
	[-0.029,0.125]	[-0.061,0.044]	u_a8_p	0.217
u_a9	0.084**	-0.003	u_a9_chi2	3.613
	[0.006,0.163]	[-0.057,0.050]	u_a9_p	0.057
u_a10	0.085**	-0.004	u_a10_chi2	3.674
	[0.006,0.164]	[-0.058,0.049]	u_a10_p	0.055
Control	Y	Y		
N	9,651	9,094		
R-sq	0.164	0.115		

Columns (1) and (2) show the impact of having a child on employment (equals to 1 if employed) for men and women, respectively. The independent variables are event time dummies, indexed such that u_bt means t years before the year of arrival of first child, and u_at means t years after the year of arrival of the first child, respectively. The event-time dummy at t = -1 is omitted, implying that the event-time coefficients measure the impact of children relative to the year just before the first childbirth. Individual controls included years of schooling and age fixed effects to control for life cycle trends. City-fixed effects, year-fixed effects, province-year-fixed effects, the year-of-survey-fixed effects, and the month-of-survey-fixed effects are included. To test whether the coefficients by subgroups are significantly different, we use the test drawing on seemingly unrelated regressions with the command suest in Stata. *p < 0.1, **p < 0.05, and ***p < 0.01, 95% confidence intervals are in brackets.

Supplementary Table 10. Impact of childbirth on hours worked per week for women and men

	(1)	(2)	(3)	(4)
	Hours worked per week			
	Women	Men	Significance of the difference	
u_b5	0.510	3.673	u_b5_chi2	0.096
	[-16.382,17.402]	[-8.753,16.100]	u_b5_p	0.757
u_b4	-1.940	-4.483	u_b4_chi2	0.374
	[-7.733,3.854]	[-10.424,1.457]	u_b4_p	0.541
u_b3	2.145	-4.132	u_b3_chi2	2.727
	[-3.007,7.297]	[-9.793,1.529]	u_b3_p	0.099
u_b2	1.260	0.860	u_b2_chi2	0.013
	[-3.623,6.143]	[-4.153,5.872]	u_b2_p	0.908
u_a0	-2.566	-0.139	u_a0_chi2	1.157
	[-5.978,0.846]	[-3.123,2.845]	u_a0_p	0.282
u_a1	-2.959*	-0.042	u_a1_chi2	1.810
	[-6.252,0.335]	[-2.904,2.819]	u_a1_p	0.179
u_a2	-2.542	-0.420	u_a2_chi2	0.987
	[-5.737,0.653]	[-3.326,2.486]	u_a2_p	0.320
u_a3	1.397	0.522	u_a3_chi2	0.163
	[-1.678,4.472]	[-2.414,3.457]	u_a3_p	0.686
u_a4	1.205	1.804	u_a4_chi2	0.076
	[-1.985,4.396]	[-1.223,4.832]	u_a4_p	0.783
u_a5	1.507	1.074	u_a5_chi2	0.038
	[-1.756,4.770]	[-2.011,4.158]	u_a5_p	0.845
u_a6	1.217	1.315	u_a6_chi2	0.002
	[-2.124,4.558]	[-1.835,4.466]	u_a6_p	0.965
u_a7	0.631	2.124	u_a7_chi2	0.432
	[-2.748,4.011]	[-1.010,5.258]	u_a7_p	0.511
u_a8	3.572**	2.356	u_a8_chi2	0.271
	[0.080,7.065]	[-0.866,5.577]	u_a8_p	0.602
u_a9	0.234	0.133	u_a9_chi2	0.002
	[-3.343,3.812]	[-3.157,3.423]	u_a9_p	0.966
u_a10	2.378	1.465	u_a10_chi2	0.141
	[-1.303,6.060]	[-1.875,4.805]	u_a10_p	0.707
Control	Y	Y		
N	5,656	7,368		
R^2	0.165	0.135		

Columns (1) and (2) show the impact of having a child on hours worked per week for men and women, respectively. The independent variables are event time dummies, indexed such that u_bt means t years before the year of arrival of first child, and u_at means t years after the year of arrival of the first child, respectively. The event-time dummy at t = -1 is omitted, implying that the event-time coefficients measure the impact of children relative to the year just before the first childbirth. Individual controls included years of schooling and age fixed effects to control for life cycle trends. City-fixed effects, year-fixed effects, province-year-fixed effects, the year-of-survey-fixed effects, and the month-of-survey-fixed effects are included. To test whether the coefficients by subgroups are significantly different, we use the test drawing on seemingly unrelated regressions with the command suest in Stata. *p < 0.1, **p < 0.05, and ***p < 0.01, 95% confidence intervals are in brackets.

Supplementary Table 11. Impact of childbirth on income for women and men

	(1)	(2)	(3)	(4)
	Income			
	Women	Men	Significance of the difference	
u_b5	-0.520*	0.338	u_b5_chi2	2.464
	[-1.100,0.060]	[-0.579,1.255]	u_b5_p	0.116
u_b4	-0.168	-0.181	u_b4_chi2	0.001
	[-0.626,0.291]	[-0.722,0.360]	u_b4_p	0.970
u_b3	0.048	0.163	u_b3_chi2	0.071
	[-0.516,0.612]	[-0.531,0.856]	u_b3_p	0.790
u_b2	0.057	0.007	u_b2_chi2	0.042
	[-0.285,0.399]	[-0.341,0.356]	u_b2_p	0.837
u_a0	-0.328***	0.330***	u_a0_chi2	16.830
	[-0.540,-0.116]	[0.085,0.575]	u_a0_p	0.000
u_a1	-0.565***	0.136	u_a1_chi2	19.511
	[-0.764,-0.367]	[-0.094,0.367]	u_a1_p	0.000
u_a2	-0.424***	0.330**	u_a2_chi2	21.134
	[-0.642,-0.205]	[0.077,0.583]	u_a2_p	0.000
u_a3	-0.436***	0.366***	u_a3_chi2	24.084
	[-0.650,-0.223]	[0.118,0.614]	u_a3_p	0.000
u_a4	-0.405***	0.318**	u_a4_chi2	18.579
	[-0.628,-0.182]	[0.058,0.577]	u_a4_p	0.000
u_a5	-0.349***	0.246*	u_a5_chi2	12.454
	[-0.575,-0.123]	[-0.017,0.509]	u_a5_p	0.000
u_a6	-0.346***	0.420***	u_a6_chi2	19.753
	[-0.580,-0.113]	[0.153,0.687]	u_a6_p	0.000
u_a7	-0.421***	0.273**	u_a7_chi2	15.769
	[-0.658,-0.183]	[0.003,0.544]	u_a7_p	0.000
u_a8	-0.309**	0.393***	u_a8_chi2	15.605
	[-0.552,-0.067]	[0.117,0.669]	u_a8_p	0.000
u_a9	-0.532***	0.235	u_a9_chi2	18.040
	[-0.779,-0.285]	[-0.049,0.520]	u_a9_p	0.000
u_a10	-0.485***	0.261*	u_a10_chi2	17.577
	[-0.733,-0.238]	[-0.016,0.538]	u_a10_p	0.000
Controls	Y	Y		
N	9,978	9,752		
R-sq	0.433	0.335		

Columns (1) and (2) show the impact of having a child on income (10,000 Yuan) men and women, respectively. The independent variables are event time dummies, indexed such that u_bt means t years before the year of arrival of first child, and u_at means t years after the year of arrival of the first child, respectively. The event-time dummy at t = -1 is omitted, implying that the event-time coefficients measure the impact of children relative to the year just before the first childbirth. Individual controls included years of schooling and age fixed effects to control for life cycle trends. City-fixed effects, year-fixed effects, province-year-fixed effects, the year-of-survey-fixed effects, and the month-of-survey-fixed effects are included. To test whether the coefficients by subgroups are significantly different, we use the test drawing on seemingly unrelated regressions with the command suest in Stata. *p < 0.1, **p < 0.05, and ***p < 0.01, 95% confidence intervals are in brackets.

Supplementary Table 12. Impact of childbirth on hourly wage for women and men

	(1)	(2)	(3)	(4)
	Hourly wage		Significance of difference	
	Female	Male		
u_b5	-2.230	11.499	u_b5_chi2	1.431
	[-21.409,16.950]	[-2.563,25.561]	u_b5_p	0.232
u_b4	-3.901	0.217	u_b4_chi2	0.762
	[-9.507,1.706]	[-7.545,7.980]	u_b4_p	0.383
u_b3	-0.431	13.000*	u_b3_chi2	2.810
	[-8.176,7.313]	[-1.231,27.232]	u_b3_p	0.094
u_b2	-1.030	2.427	u_b2_chi2	0.886
	[-6.151,4.092]	[-2.892,7.747]	u_b2_p	0.347
u_a0	-3.088	3.178	u_a0_chi2	3.810
	[-7.713,1.537]	[-1.336,7.693]	u_a0_p	0.051
u_a1	-4.748*	-0.461	u_a1_chi2	1.315
	[-9.788,0.292]	[-5.799,4.877]	u_a1_p	0.252
u_a2	-4.800*	-0.020	u_a2_chi2	1.094
	[-10.211,0.610]	[-7.675,7.635]	u_a2_p	0.296
u_a3	-1.498	1.211	u_a3_chi2	0.373
	[-8.629,5.633]	[-4.235,6.657]	u_a3_p	0.541
u_a4	-4.364	-1.885	u_a4_chi2	0.232
	[-9.897,1.169]	[-10.846,7.077]	u_a4_p	0.630
u_a5	-4.373	10.362	u_a5_chi2	1.685
	[-9.599,0.852]	[-11.695,32.420]	u_a5_p	0.194
u_a6	-5.986**	-3.443	u_a6_chi2	0.130
	[-10.913,-1.059]	[-16.900,10.015]	u_a6_p	0.718
u_a7	-3.721	-4.572	u_a7_chi2	0.013
	[-8.992,1.550]	[-18.898,9.754]	u_a7_p	0.910
u_a8	-5.412**	18.394	u_a8_chi2	3.000
	[-10.799,-0.026]	[-8.750,45.537]	u_a8_p	0.083
u_a9	-5.820*	-0.666	u_a9_chi2	0.704
	[-11.797,0.157]	[-11.658,10.325]	u_a9_p	0.401
u_a10	3.179	0.524	u_a10_chi2	0.051
	[-16.234,22.592]	[-13.012,14.060]	u_a10_p	0.822
Control	Y	Y		
N	5,180	6,912		
R^2	0.073	0.035		

Columns (1) and (2) show the results of event study for men and women, respectively. The dependent variables in both columns are hourly wage. The independent variables are event time dummies, indexed such that u_bt mean t years before the year of arrival of first child, and u_at mean t years after the year of arrival of the first child, respectively. The event-time dummy at t = -1 was omitted, implying that the event-time coefficients measure the impact of children relative to the year just before the first childbirth. Individual controls included years of schooling and age fixed effects to control for life cycle trends. City-fixed effects, year-fixed effects, province-year-fixed effects, the year-of-survey-fixed effects, and the month-of-survey-fixed effects are included. To test whether the coefficients by subgroups are significantly different, we use the test drawing on seemingly unrelated regressions with the command suest in Stata. *p < 0.1, **p < 0.05, and ***p < 0.01, 95% confidence intervals are in brackets.

Supplementary Table 13. Heterogeneity by region (urban or rural areas)

	(1)<br>Urban	(2)<br>Rural
u_b5	-0.388	0.860***
	[-1.270,0.493]	[0.409,1.312]
u_b4	-0.221	-0.219
	[-0.519,0.077]	[-0.707,0.268]
u_b3	-0.055	-0.254
	[-0.467,0.358]	[-0.768,0.260]
u_b2	-0.162	0.069
	[-0.414,0.089]	[-0.199,0.337]
u_a0	-0.156*	-0.041
	[-0.332,0.021]	[-0.221,0.140]
u_a1	-0.273***	-0.002
	[-0.437,-0.108]	[-0.179,0.174]
u_a2	-0.240***	-0.120
	[-0.408,-0.072]	[-0.302,0.061]
u_a3	-0.319***	-0.018
	[-0.485,-0.153]	[-0.205,0.168]
u_a4	-0.212**	-0.092
	[-0.384,-0.041]	[-0.276,0.091]
u_a5	-0.275***	-0.093
	[-0.449,-0.102]	[-0.286,0.100]
u_a6	-0.275***	-0.083
	[-0.456,-0.094]	[-0.274,0.108]
u_a7	-0.227**	-0.057
	[-0.409,-0.045]	[-0.257,0.142]
u_a8	-0.259***	-0.054
	[-0.444,-0.073]	[-0.256,0.148]
u_a9	-0.189*	-0.041
	[-0.380,0.001]	[-0.246,0.165]
u_a10	-0.223**	-0.064
	[-0.418,-0.028]	[-0.270,0.142]
Control	Y	Y
N	5,706	5,686
R-sq	0.124	0.132

Columns (1) and (2) show the results of event study for women in urban and rural areas, respectively. The independent variables are event time dummies, indexed such that u_bt means t years before the year of arrival of first child, and u_at means t years after the year of arrival of the first child, respectively. The event-time dummy at t = -1 is omitted, implying that the event-time coefficients measure the impact of children relative to the year just before the first childbirth. Individual controls included years of schooling and age fixed effects to control for life cycle trends. City-fixed effects, year-fixed effects, province-year-fixed effects, the year-of-survey-fixed effects, and the month-of-survey-fixed effects are included. *p < 0.1, **p < 0.05, and ***p < 0.01, 95% confidence intervals are in brackets.

Supplementary Table 14. Heterogeneity by income group

	(1)<br>Higher income	(2)<br>Lower income
u_b5	0.241	0.027
	[-0.599,1.080]	[-0.903,0.957]
u_b4	-0.547***	0.034
	[-0.875,-0.219]	[-0.379,0.447]
u_b3	0.022	-0.225
	[-0.456,0.500]	[-0.637,0.186]
u_b2	-0.139	0.049
	[-0.404,0.126]	[-0.220,0.318]
u_a0	-0.161	-0.081
	[-0.359,0.037]	[-0.259,0.097]
u_a1	-0.195**	-0.084
	[-0.389,-0.001]	[-0.256,0.088]
u_a2	-0.276***	-0.113
	[-0.472,-0.081]	[-0.291,0.065]
u_a3	-0.160*	-0.126
	[-0.349,0.028]	[-0.307,0.055]
u_a4	-0.250***	-0.102
	[-0.440,-0.060]	[-0.287,0.083]
u_a5	-0.342***	-0.116
	[-0.537,-0.146]	[-0.305,0.074]
u_a6	-0.278***	-0.134
	[-0.478,-0.078]	[-0.328,0.059]
u_a7	-0.275***	-0.085
	[-0.477,-0.072]	[-0.284,0.113]
u_a8	-0.238**	-0.042
	[-0.448,-0.028]	[-0.242,0.159]
u_a9	-0.203*	0.030
	[-0.424,0.017]	[-0.173,0.232]
u_a10	-0.270**	-0.080
	[-0.494,-0.047]	[-0.283,0.123]
Control	Y	Y
N	3,330	6,082
R-sq	0.159	0.114

Columns (1) and (2) show the results of event study for women in high-income and low-income groups, respectively. The independent variables are event time dummies, indexed such that u_bt means t years before the year of arrival of first child, and u_at means t years after the year of arrival of the first child, respectively. The event-time dummy at t = -1 is omitted, implying that the event-time coefficients measure the impact of children relative to the year just before the first childbirth. Individual controls included years of schooling and age fixed effects to control for life cycle trends. City-fixed effects, year-fixed effects, province-year-fixed effects, the year-of-survey-fixed effects, and the month-of-survey-fixed effects are included. 95% confidence intervals are in brackets, with *p < 0.1, **p < 0.05, and ***p < 0.01.

Supplementary Table 15. Heterogeneity by education

	(1)<br>Higher education	(1)<br>Lower education
u_b5	0.564	-0.026
	[-0.297,1.425]	[-1.132,1.079]
u_b4	-0.079	-0.308
	[-0.423,0.265]	[-0.719,0.102]
u_b3	-0.108	-0.180
	[-0.543,0.327]	[-0.643,0.283]
u_b2	-0.140	0.111
	[-0.378,0.099]	[-0.179,0.401]
u_a0	-0.182**	-0.038
	[-0.359,-0.004]	[-0.214,0.139]
u_a1	-0.133	-0.116
	[-0.300,0.034]	[-0.288,0.056]
u_a2	-0.208**	-0.156*
	[-0.381,-0.036]	[-0.334,0.021]
u_a3	-0.172*	-0.153*
	[-0.348,0.004]	[-0.332,0.025]
u_a4	-0.208**	-0.116
	[-0.388,-0.029]	[-0.295,0.062]
u_a5	-0.156	-0.180*
	[-0.342,0.030]	[-0.364,0.004]
u_a6	-0.180*	-0.159*
	[-0.369,0.009]	[-0.346,0.029]
u_a7	-0.157	-0.134
	[-0.358,0.044]	[-0.323,0.055]
u_a8	-0.169	-0.126
	[-0.371,0.033]	[-0.319,0.066]
u_a9	-0.126	-0.069
	[-0.335,0.082]	[-0.263,0.125]
u_a10	-0.094	-0.140
	[-0.308,0.120]	[-0.336,0.057]
Control	Y	Y
N	4,189	7,325
R-sq	0.121	0.122

Columns (1) and (2) show the results of event study for women with high educational attainment and low educational attainment, respectively. The independent variables are event time dummies, indexed such that u_bt means t years before the year of arrival of first child, and u_at means t years after the year of arrival of the first child, respectively. The event-time dummy at t = -1 is omitted, implying that the event-time coefficients measure the impact of children relative to the year just before the first childbirth. Individual controls included years of schooling and age fixed effects to control for life cycle trends. City-fixed effects, year-fixed effects, province-year-fixed effects, the year-of-survey-fixed effects, and the month-of-survey-fixed effects are included. 95% confidence intervals are in brackets, with *p < 0.1, **p < 0.05, and ***p < 0.01.

Supplementary Table 16. Heterogeneity by age

	(1)<br>Age > 27	(2)<br>Age <= 27
u_b5	-1.044**	0.454
	[-2.062,-0.027]	[-0.140,1.049]
u_b4	-0.134	-0.254
	[-0.569,0.301]	[-0.594,0.086]
u_b3	0.016	-0.153
	[-0.645,0.677]	[-0.514,0.207]
u_b2	-0.073	-0.044
	[-0.445,0.298]	[-0.258,0.170]
u_a0	-0.157	-0.118
	[-0.407,0.093]	[-0.262,0.026]
u_a1	-0.168	-0.143**
	[-0.408,0.072]	[-0.285,-0.002]
u_a2	-0.175	-0.235***
	[-0.402,0.053]	[-0.382,-0.088]
u_a3	-0.160	-0.207***
	[-0.381,0.061]	[-0.360,-0.055]
u_a4	-0.184	-0.182**
	[-0.403,0.035]	[-0.343,-0.021]
u_a5	-0.137	-0.280***
	[-0.357,0.083]	[-0.456,-0.104]
u_a6	-0.185*	-0.160
	[-0.404,0.035]	[-0.355,0.034]
u_a7	-0.143	-0.183
	[-0.363,0.077]	[-0.415,0.050]
u_a8	-0.144	-0.200
	[-0.364,0.076]	[-0.527,0.127]
u_a9	-0.087	-0.346
	[-0.308,0.134]	[-0.856,0.163]
u_a10	-0.144	0.055
	[-0.367,0.079]	[-0.661,0.771]
Control	Y	Y
N	7,203	4,317
R-sq	0.116	0.119

Columns (1) and (2) show the results of event study for women in younger and older groups, respectively. The independent variables are event time dummies, indexed such that u_bt means t years before the year of arrival of first child, and u_at means t years after the year of arrival of the first child, respectively. The event-time dummy at t = -1 is omitted, implying that the event-time coefficients measure the impact of children relative to the year just before the first childbirth. Individual controls included years of schooling and age fixed effects to control for life cycle trends. City-fixed effects, year-fixed effects, province-year-fixed effects, the year-of-survey-fixed effects, and the month-of-survey-fixed effects are included. 95% confidence intervals are in brackets, with *p < 0.1, **p < 0.05, and ***p < 0.01.

Supplementary Table 17. Heterogeneity by whether children were primarily looked after by mother or not

	(1)<br>Children taken care of by mom	(2)<br>Children taken care of by others
u_b5	1.175***	
	[0.638,1.713]	
u_b4	-0.389	-0.933**
	[-1.334,0.556]	[-1.867,-0.000]
u_b2	0.024	-0.742*
	[-0.692,0.740]	[-1.525,0.041]
u_a0	-0.250	0.097
	[-0.588,0.088]	[-0.447,0.640]
u_a1	-0.257	-0.227
	[-0.595,0.082]	[-0.748,0.294]
u_a2	-0.285*	-0.330
	[-0.622,0.051]	[-0.852,0.193]
u_a3	-0.300*	-0.267
	[-0.636,0.037]	[-0.789,0.254]
u_a4	-0.282	-0.281
	[-0.621,0.056]	[-0.798,0.236]
u_a5	-0.305*	-0.219
	[-0.647,0.037]	[-0.738,0.301]
u_a6	-0.321*	-0.220
	[-0.663,0.020]	[-0.745,0.304]
u_a7	-0.293*	-0.170
	[-0.637,0.051]	[-0.706,0.366]
u_a8	-0.266	-0.261
	[-0.610,0.077]	[-0.793,0.271]
u_a9	-0.231	-0.224
	[-0.577,0.115]	[-0.756,0.309]
u_a10	-0.288	-0.235
	[-0.635,0.059]	[-0.776,0.307]
Control	Y	Y
N	8,182	2,201
R-sq	0.102	0.210

Columns (1) and (2) show the results of event study for women whose children were primarily looked after by themselves and those not, respectively. The independent variables are event time dummies, indexed such that u_bt means t years before the year of arrival of first child, and u_at means t years after the year of arrival of the first child, respectively. The event-time dummy at $t = -1$ is omitted, implying that the event-time coefficients measure the impact of children relative to the year just before the first childbirth. Individual controls included years of schooling and age fixed effects to control for life cycle trends. City-fixed effects, year-fixed effects, province-year-fixed effects, the year-of-survey-fixed effects, and the month-of-survey-fixed effects are included. 95% confidence intervals are in brackets, with *$p < 0.1$, **$p < 0.05$, and ***$p < 0.01$.

Supplementary Table 18. Heterogeneity by reproductive age

	(1)<br>Firstborn > 24	(2)<br>Firstborn <= 24
u_b5	0.050	
	[-0.671,0.772]	
u_b4	-0.244*	0.183
	[-0.525,0.037]	[-1.188,1.555]
u_b3	-0.179	0.315
	[-0.540,0.182]	[-0.258,0.889]
u_b2	-0.039	0.129
	[-0.262,0.183]	[-0.277,0.535]
u_a0	-0.068	-0.202**
	[-0.242,0.107]	[-0.393,-0.012]
u_a1	-0.097	-0.263***
	[-0.265,0.071]	[-0.450,-0.077]
u_a2	-0.117	-0.341***
	[-0.290,0.056]	[-0.538,-0.144]
u_a3	-0.117	-0.329***
	[-0.295,0.061]	[-0.533,-0.125]
u_a4	-0.173*	-0.299***
	[-0.357,0.010]	[-0.508,-0.091]
u_a5	-0.119	-0.378***
	[-0.310,0.072]	[-0.596,-0.160]
u_a6	-0.153	-0.360***
	[-0.346,0.040]	[-0.589,-0.132]
u_a7	-0.094	-0.338***
	[-0.296,0.109]	[-0.575,-0.102]
u_a8	-0.139	-0.334***
	[-0.346,0.068]	[-0.581,-0.087]
u_a9	-0.129	-0.230*
	[-0.339,0.082]	[-0.493,0.033]
u_a10	-0.185*	-0.272**
	[-0.400,0.031]	[-0.543,-0.001]
Control	Y	Y
N	5,041	6,475
R-sq	0.118	0.118

Columns (1) and (2) show the results of event study for women who have the first child before the age of 24 years and after, respectively. The independent variables are event time dummies, indexed such that u_bt means t years before the year of arrival of first child, and u_at means t years after the year of arrival of the first child, respectively. The event-time dummy at t = -1 is omitted, implying that the event-time coefficients measure the impact of children relative to the year just before the first childbirth. Individual controls included years of schooling and age fixed effects to control for life cycle trends. City fixed effects, year fixed effects, province year fixed effects, the year of survey fixed effects, and the month of survey fixed effects are included. 95% confidence intervals are in brackets, with *p < 0.1, **p < 0.05, and ***p < 0.01.

Supplementary Table 19. Heterogeneity by whether having a son

	(1)<br>without a son	(2)<br>with a son
u_b5	0.507	0.015
	[-0.403,1.416]	[-0.860,0.889]
u_b4	-0.074	-0.346**
	[-0.510,0.363]	[-0.684,-0.009]
u_b3	-0.207	-0.061
	[-0.673,0.259]	[-0.480,0.358]
u_b2	-0.143	0.024
	[-0.440,0.154]	[-0.202,0.251]
u_a0	-0.155	-0.122
	[-0.363,0.053]	[-0.281,0.036]
u_a1	-0.112	-0.200***
	[-0.311,0.088]	[-0.351,-0.048]
u_a2	-0.108	-0.285***
	[-0.314,0.098]	[-0.441,-0.129]
u_a3	-0.201*	-0.208***
	[-0.415,0.012]	[-0.363,-0.054]
u_a4	-0.199*	-0.212***
	[-0.414,0.016]	[-0.369,-0.055]
u_a5	-0.292**	-0.217***
	[-0.522,-0.063]	[-0.377,-0.057]
u_a6	-0.177	-0.256***
	[-0.415,0.060]	[-0.418,-0.095]
u_a7	-0.108	-0.238***
	[-0.346,0.130]	[-0.405,-0.072]
u_a8	-0.132	-0.228***
	[-0.378,0.115]	[-0.396,-0.059]
u_a9	-0.099	-0.175**
	[-0.348,0.151]	[-0.346,-0.003]
u_a10	-0.202	-0.209**
	[-0.466,0.062]	[-0.381,-0.036]
Control	Y	Y
N	3,255	8,264
R^2	0.163	0.104

This table shows the results of event study for women without and with sons, respectively. The independent variables are event time dummies, indexed such that u_bt means t years before the year of arrival of first child, and u_at means t years after the year of arrival of the first child, respectively. The event-time dummy at t = -1 is omitted, implying that the event-time coefficients measure the impact of children relative to the year just before the first childbirth. Individual controls included years of schooling and age fixed effects to control for life cycle trends. City fixed effects, year fixed effects, province year fixed effects, the year of survey fixed effects, and the month of survey fixed effects are included. 95% confidence intervals are in brackets, with *p < 0.1, **p < 0.05, and ***p < 0.01.

Supplementary Table 20. Heterogeneity by the number of children

	(1)	(2)
Female	One child	Multiple children
u_b5	1.124	-0.008
	[-0.297,2.545]	[-0.678,0.662]
u_b4	-0.208	-0.240
	[-0.650,0.234]	[-0.570,0.090]
u_b3	0.165	-0.265
	[-0.430,0.759]	[-0.618,0.088]
u_b2	-0.168	-0.008
	[-0.567,0.231]	[-0.217,0.201]
u_a0	-0.064	-0.118
	[-0.331,0.203]	[-0.259,0.023]
u_a1	-0.079	-0.162**
	[-0.336,0.178]	[-0.295,-0.028]
u_a2	-0.118	-0.223***
	[-0.392,0.157]	[-0.359,-0.086]
u_a3	-0.117	-0.207***
	[-0.390,0.155]	[-0.344,-0.070]
u_a4	-0.077	-0.210***
	[-0.355,0.201]	[-0.349,-0.072]
u_a5	-0.097	-0.233***
	[-0.375,0.181]	[-0.378,-0.089]
u_a6	-0.075	-0.242***
	[-0.363,0.213]	[-0.388,-0.096]
u_a7	-0.035	-0.216***
	[-0.329,0.259]	[-0.365,-0.066]
u_a8	-0.107	-0.184**
	[-0.397,0.183]	[-0.338,-0.030]
u_a9	-0.036	-0.158**
	[-0.335,0.263]	[-0.315,-0.001]
u_a10	-0.084	-0.201**
	[-0.382,0.213]	[-0.362,-0.039]
Control	Y	Y
N	2,807	8,704
R^2	0.147	0.112

This table shows the results of event study for women with one and multiple children, respectively. The independent variables are event time dummies, indexed such that u_bt means t years before the year of arrival of first child, and u_at means t years after the year of arrival of the first child, respectively. The event-time dummy at t = -1 is omitted, implying that the event-time coefficients measure the impact of children relative to the year just before the first childbirth. Individual controls included years of schooling and age fixed effects to control for life cycle trends. City fixed effects, year fixed effects, province year fixed effects, the year of survey fixed effects, and the month of survey fixed effects are included. 95% confidence intervals are in brackets, with *p < 0.1, **p < 0.05, and ***p < 0.01.

Supplementary Table 21. Impact of childbirth on life satisfaction and mediators for women

	(1)	(2)	(3)	(4)	(5)	(6)
		Economic/utilitarian		Psychological/emotional		Physical factor
	Life Satisfaction	Financial support	Income	Co-residence with parents	Social status	Improved physical health
after	-0.132***	0.025	-0.439***	0.037	0.003	-0.037**
	[-0.22,-0.05]	[-0.04,0.09]	[-0.59,-0.28]	[-0.01,0.08]	[-0.08,0.09]	[-0.07,-0.01]
Control	Y	Y	Y	Y	Y	Y
N	11,520	4,887	9,425	7,980	11,490	11,514
R^2	0.095	0.083	0.452	0.132	0.063	0.046

the results show the impact of childbirth on life satisfaction and mediators for women. In column 2, the dependent variable is financial support, which takes 1 when get economic support from parents, otherwise, it takes 0. The dependent variable in column 3 is personal income. In column 4, the co-residence with parents takes 1 when live with parents, otherwise, it takes 0. The dependent variable in column 5 is self-reported social status ranging from 1 to 5, with the higher the score representing higher social status. The dependent variable in column 6 is improved physical health, which takes 1 when having a better health, otherwise, it is 0. The independent variable of interest "after" is a time dummy. It takes 1 when the time is after the arrival of first child; 0, otherwise. The control variables are the same as in Table 2. 95% confidence intervals are in brackets, with *p < 0.1, **p < 0.05, and ***p < 0.01.

Supplementary Table 22. Impact of childbirth on life satisfaction with mediators included

	(1)	(2)	(3)	(4)	(5)
			Life satisfaction		
after	-0.095	-0.112**	-0.106*	-0.132***	-0.124***
	[-0.22,0.03]	[-0.20,-0.02]	[-0.21,0.00]	[-0.22,-0.05]	[-0.21,-0.04]
Financial support	-0.100***				
	[-0.17,0.03]				
Income		0.012*			
		[-0.00,0.03]			
Co-residence with parents			0.028		
			[-0.04,0.09]		
Social status				0.281***	
				[0.26,0.30]	
Improved physical health					0.200***
					[0.14,0.26]
Control	Y	Y	Y	Y	Y
N	4,887	9,425	7,980	11,490	11,514
R^2	0.093	0.103	0.093	0.164	0.098

This table shows the impact of childbirth on life satisfaction with mediators included. The variable financial support is equal to 1 if women receive financial supports from parents, 0, otherwise. The variable co-residence is equal to 1 if women live with parents, 0, otherwise. The variable self-reported social status, ranges from 1 to 5, with the higher the score representing the higher social status. The variable improved physical health is equal to 1 when having a better health, 0, otherwise. The independent variable of interest "after" is a time dummy, which is equal to 1 when the time is after the arrival of first child; 0, otherwise. The control variables are the same as in Table 2. 95% confidence intervals are in brackets, with *p < 0.1, **p < 0.05, and ***p < 0.01.
